# Structure determination from unindexed powder data from scratch by a global optimization approach using pattern comparison based on cross-correlation functions

**DOI:** 10.1107/S2052520622001500

**Published:** 2022-03-16

**Authors:** Stefan Habermehl, Carina Schlesinger, Martin U. Schmidt

**Affiliations:** a Institute of Inorganic and Analytical Chemistry, Goethe University, Max-von-Laue-Strasse 7, 60438 Frankfurt am Main, Germany

**Keywords:** structure determination from powder diffraction data, global optimization, cross-correlation functions, unindexed powder patterns, fit with deviating unit-cell parameters

## Abstract

A method of *ab initio* crystal structure determination from powder diffraction data for organic and metal–organic compounds, which does not require prior indexing of the powder pattern, has been developed. Only a reasonable molecular geometry is required, needing knowledge of neither unit-cell parameters nor space group. The structures are solved from scratch by a global fit to the powder data using the new program *FIDEL-GO* (‘FIt with DEviating Lattice parameters - Global Optimization’). *FIDEL-GO* uses a similarity measure based on cross-correlation functions, which allows the comparison of simulated and experimental powder data even if the unit-cell parameters deviate strongly. The optimization starts from large sets of random structures in various space groups. The unit-cell parameters, molecular position and orientation, and selected internal degrees of freedom are fitted simultaneously to the powder pattern. The optimization proceeds in an elaborate multi-step procedure with built-in clustering of duplicate structures and iterative adaptation of parameter ranges. The best structures are selected for an automatic Rietveld refinement. Finally, a user-controlled Rietveld refinement is performed. The procedure aims for the analysis of a wide range of ‘problematic’ powder patterns, in particular powders of low crystallinity. The method can also be used for the clustering and screening of a large number of possible structure candidates and other application scenarios. Examples are presented for structure determination from unindexed powder data of the previously unknown structures of the nanocrystalline phases of 4,11-difluoro-, 2,9-dichloro- and 2,9-dichloro-6,13-dihydro-quinacridone, which were solved from powder patterns with 14–20 peaks only, and of the coordination polymer dichloro-bis(pyridine-*N*)copper(II).

## Abstract 2

A new method for the structure determination of molecular crystals from unindexed powder data has been developed and successfully applied. The method performs a global optimization using pattern comparison based on cross-correlation functions.

## 1. Introduction

Structure determination from powder diffraction data (SDPD) is an important technique for the investigation of crystalline solids (David *et al.*, 2002 ▸; David & Shankland, 2008 ▸; Harris, 2012 ▸; Černý, 2017 ▸). This is particularly true if the material can not be prepared as a single crystal, or in cases where the structure of a powder of low crystallinity is at the centre of interest. SDPD generally starts with the indexing of the powder pattern. Reliable indexing fails if the pattern contains too few or too broad reflections or if the powder is not phase-pure (Brüning & Schmidt, 2015 ▸).

If indexing fails, the intensities of the *hkl* reflections cannot be extracted and reciprocal-space approaches such as direct methods cannot be applied. Also, the common direct-space methods that solve structures by translation, rotation and conformational changes of the molecules in the unit cell require the unit-cell parameters as input, *e.g.* in *DASH* (David *et al.*, 2006 ▸; Spillman *et al.*, 2015 ▸), *FOX* (Favre-Nicolin & Černý, 2004 ▸; Černý *et al.*, 2017 ▸), *EXPO* (Altomare *et al.*, 2009 ▸, 2013 ▸), *TOPAS* (Coelho, 2018 ▸) or *MRIA* (Zhukov *et al.*, 2001 ▸).

Without knowledge of the unit-cell parameters and space group there are two main obstacles:

(i) Six additional parameters (*a*, *b*, *c*, α, β, γ) must be determined. This is not a principal problem, because they correspond to the reflection positions. However, it implies an enormous expansion of the search space and an increase in the required computing time.

(ii) The exploration of the search space depends on comparison of the observed pattern with the powder patterns simulated from structural models. Common direct-space methods and Rietveld refinement perform this comparison based on pointwise differences between the two curves, *i.e.* the intensity differences at each individual 2θ value. This approach to quantifying the agreement between the patterns, *e.g.* by the most commonly used χ^2^ and *R*_wp_ values (Toby, 2006 ▸), only works for changes in atomic coordinates, and for small changes in the unit-cell parameters, if the reflection positions do not shift by more than a few reflection half widths. The powder pattern is highly sensitive to even small changes in the unit-cell parameters. Hence, the comparative measure becomes meaningless if the unit-cell parameters of the structural model deviate too much from the correct ones and the simulated reflections do not overlap with the corresponding signals in the observed pattern.

In contrast to the intense development of global optimization methods working within the subspace determined by a given unit cell, there have been only a few attempts to apply global optimization approaches to the extensive global space beyond that. The search space contains more parameters, *i.e.* more dimensions, which are difficult to track. The even more crucial element is the link between model and experiment. The shape and characteristics of the multidimensional (dis)similarity hypersurface substantially affect the effectiveness and efficiency of the global search and local optimizations. Attempts at SDPD without prior indexing typically address these problems using alternative approaches to pattern comparison or using energy calculations. Hofmann & Kuleshova (2006 ▸) used a similarity index based on the distances between the normalized integral curves of the patterns for structure fitting to the powder data, starting from crystal structures predicted by force-field lattice energy minimization. Padgett *et al.* (2007 ▸) employed a combination of grid search and a genetic algorithm in the program *OCEANA*, using a combination of *R*_wp_ values and force-field energy. Rapallo (2009 ▸) developed a hybrid Monte Carlo method implemented in the software *VARICELLA*, where coordinate changes are performed according to molecular dynamics, using a joint probability density of the potential energy and a disagreement factor that compares the Fourier transforms of the patterns. De Gelder and co-workers employed a genetic algorithm for simultaneous indexing and structure solution, using pattern matching based on cross-correlation functions in the program *FIDDLE* (de Gelder *et al.*, 2008 ▸; Guguta, 2009 ▸; Smits *et al.*, 2009 ▸). However, all these approaches have their limitations, and to our knowledge there is at present no generally applicable well functioning method for the structure determination of molecular crystals from unindexable powder data.

We developed a robust local optimization procedure that uses pattern matching based on cross-correlation functions for the fitting of a structural model to the experimental pattern. The approach was first implemented in the computer program *FIDEL* (FIt with DEviating Lattice parameters) (Habermehl *et al.*, 2014 ▸). *FIDEL* fits not only the unit-cell parameters, but also simultaneously the position and orientation of the molecules and selected internal degrees of freedom. *FIDEL* proved to be capable of fitting significantly deviating structural models to powder data of high or low quality. In a previous paper (Habermehl *et al.*, 2014 ▸), we described the useful and successful application of the procedure to the refinement of crystal structures, if a suitable structural model with possibly strongly deviating unit-cell parameters is available. The model may come from the crystal structures of isostructural compounds (*e.g.* solvates, hydrates or chemical derivatives), or from diffraction data measured at a different temperature or pressure. Alternatively, trial models can be obtained by a crystal structure prediction (CSP), *e.g.* a global lattice energy minimization. Typically, the simulated powder patterns of these structural models deviate significantly from the experimental data, in particular in their reflection positions. Nevertheless, the *FIDEL* fits were successful.

### 1.1. The generalized expression for similarity

To compare powder patterns and fit a crystal structure to the experimental powder pattern, *FIDEL* uses the generalized similarity measure *S*_12_, which was introduced by de Gelder *et al.* (2001 ▸) as a versatile similarity criterion for pattern comparison. *S*_12_ correlates data points within a certain 2θ neighbouring range. This absolute and normalized measure is based on the weighted cross- and auto-correlation functions of the patterns to be compared. *S*_12_ puts emphasis on the strong reflections, while being tolerant of changes in the position or shape of the reflections. It can properly recognize even rough matches, in particular with respect to signal shifts in otherwise similar patterns.

The cross-correlation function *c*_12_(*r*) of two powder patterns *I*_1_(2θ) and *I*_2_(2θ) correlates every data point of one pattern to data points at the 2θ distance of *r* in the other pattern,

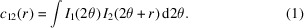




The auto-correlation functions *c*_11_(*r*) and *c*_22_(*r*) of each pattern are defined analogously. The correlation of data points, however, is restricted to a certain neighbourhood by the introduction of the triangular weighting function *w*(*r*), 

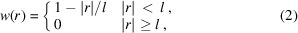

with the neighbouring range parameter *l* (*l* > 0) corresponding to the full width at half-maximum of the weighting function.

Integration of the weighted cross-correlation function leads to a single value. This value is normalized using the corresponding weighted auto-correlation functions of the two patterns, resulting in the generalized similarity measure described by de Gelder *et al.* (2001 ▸), 

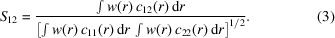




Generally, *S*_12_ can adopt values between −1 and 1. In the case of powder patterns with positive intensities, *S*_12_ adopts values between 0 and 1, where *S*_12_ = 1 corresponds to identical patterns. A schematic illustration of *S*_12_ is shown in Fig. 2 of Habermehl *et al.* (2021 ▸).

The similarity measure can be adapted to the specific characteristics of a problem by varying the neighbouring range parameter *l*. A large value of *l* allows the treatment of patterns with strongly deviating reflection positions. Narrowing the weighting function by decreasing *l*, on the other hand, leads to a more accurate comparison, which is useful for already very similar patterns. The limit of *S*_12_ as *l* approaches 0 leads to a pointwise comparison of the two diagrams that corresponds to the Pearson correlation coefficient (de Gelder *et al.*, 2001 ▸; Habermehl *et al.*, 2021 ▸). The values of *S*_12_(*l* = 0) based on the full 2θ range of the experimental data are denoted here as the reference similarities 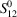 (including the background on both sides) and 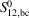 for background-corrected patterns. 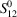 and 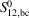 are employed as reference values for the comparison of results obtained with different values of *l* and different 2θ comparison ranges.

The similarity measure *S*_12_ can be used for:

(i) The comparison of two crystal structures, regardless of their chemical composition or crystal symmetry (Macrae *et al.*, 2008 ▸; Sacchi *et al.*, 2020 ▸).

(ii) The comparison of a structural model and an experimental powder pattern.

(iii) The comparison of two experimental powder patterns.

(iv) The selection of a peak shape function and optimization of its full width at half-maximum (FWHM), based on the comparison of an experimental powder pattern with simulated patterns derived from an arbitrary list of signal positions and intensities (see Section 3.1).

(v) Local optimization by fitting a crystal structural model with possibly strongly deviating unit-cell parameters to an experimental powder pattern (Habermehl *et al.*, 2014 ▸).

(vi) The clustering of similar structures by comparison of their simulated powder patterns (de Gelder *et al.*, 2001 ▸) or by fitting structural models to simulated patterns (this work).

(vii) Automatic peak alignment of a set of *in situ* X-ray powder diffraction patterns using the maximization of the similarity of experimental powder patterns (Guccione *et al.*, 2018 ▸).

(viii) The clustering of large lists of experimental powder patterns.

(ix) The screening of lists of structural models (*e.g.* from CSP or a database) by (*a*) comparison (de Gelder, 2006 ▸) or (*b*) fitting against an experimental powder pattern (Habermehl *et al.*, 2014 ▸; Neumann, 2016 ▸) (this work).

(x) Global optimization approaches to SDPD from scratch using (*a*) comparison (de Gelder *et al.*, 2008 ▸; Guguta, 2009 ▸; Smits *et al.*, 2009 ▸) or (*b*) comparison and local optimization of structural models (this work).

*S*_12_ can also be used to compare two pair distribution functions (PDFs) (Habermehl *et al.*, 2021 ▸). Correspondingly, the above-mentioned applications are possible either by comparing and fitting to powder patterns or by comparing and fitting to PDFs (*e.g.* Schlesinger *et al.*, 2021 ▸).

### 1.2. Crystal structure fitting

The similarity measure *S*_12_ is used by *FIDEL* for the fit of a crystal structure to a powder pattern. A structural model is described by the molecular geometry and a parameter vector. The molecular geometry is described by internal coordinates given as a *z* matrix (see Shankland, 2004 ▸). The parameter vector contains:

(i) The unit-cell parameters *a*, *b*, *c*, α, β, γ,

(ii) The fractional coordinates *m_x_*, *m_y_*, *m_z_* of an anchor point of the molecule or molecular ensemble,

(iii) The rotation angles φ_*x*_, φ_*y*_, φ_*z*_ describing the change in spatial orientation relative to the initial orientation, and

(iv) A number of internal degrees of freedom τ_*i*_ referring to distances, angles and torsions in the *z* matrix.

All these parameters can be fitted. Depending on the space group, some of them may be fixed or constrained. The internal degrees of freedom τ_*i*_ account for the variation in bond lengths, bond angles, rotation of bonds and even more complex conformational flexibilities. They are also used to model structures with more than one molecule in the asymmetric unit, including solvates and ionic compounds. The capability of this approach to model concerted conformation changes is limited. Some options may require sophisticated *z*-matrix constructions including dummy atoms.

During the fitting of a given crystal structural model (starting structure) to the powder pattern, the parameter vector is altered under maximization of the similarity *S*_12_ of the simulated powder pattern and the background-corrected experimental pattern as the cost function. The local optimization is done by a robust and customizable fitting procedure using steepest ascent, conjugate gradient or hill-climb algorithms. The best results are obtained with a modified hill-climb algorithm, although this also requires the most computing time.

The simulation of powder patterns from crystal structures is done based on a common methodology. The integral reflection intensities are computed from the crystal structure by 

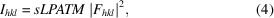

where *s* is a scaling factor, *L* the Lorentz factor, *P* the polarization factor, *A* the absorption factor, *T* a factor accounting for preferred orientation effects, *M* the reflection multiplicity according to the crystal symmetry and *F_hkl_* the complex structure factor. The powder pattern is derived by applying a peak shape function *p*(Δθ, θ) to each *I_hkl_* value in a given θ range. *FIDEL* implements different functions for *L*, *P*, *A*, *T* and *p*(Δθ, θ) that can be chosen and parametrized according to the experimental conditions of the diffraction measurement [for more details see Section 2.2 of Habermehl *et al.* (2014 ▸)].

The characteristics of the similarity measure *S*_12_ facilitate working with static inputs and settings for the modelling of the diffraction pattern that are not altered during the fitting procedure. Typical preparations include a reasonable background correction of the experimental pattern, the selection or configuration of the intensity correction functions in equation (4), the selection of a peak shape function *p*(Δθ, θ) and a raw estimate of the FWHM of the reflections. It is sufficient to satisfy these requirements once before fitting the structure. Hence, the local optimization procedure is fully focused on the fitting of the small number of structural parameters. Only the FWHM value is usually slightly adjusted at the stage of a *FIDEL* fine fit of a structural model that already matches the experimental data quite well.

*FIDEL*’s crystal structure fitting by maximization of *S*_12_ is particularly suitable for poorly crystalline compounds (broad overlapping reflections) or powder data of low quality (*e.g.* phase-impure samples, low signal-to-noise ratio). The local optimization approach has been successfully applied for automatic SDPD starting from (i) the crystal structures of isostructural compounds, (ii) crystal data measured at different temperatures, and (iii) results of CSP by force-field methods, including successful application to the powder pattern of a sample of ethyl-*tert*-butyl ether with significant phase impurity [see Section 6 of Habermehl *et al.* (2014 ▸)]. The structural models resulting from a *FIDEL* fit are subsequently refined by an automatic Pawley fit and Rietveld refinement sequence using the program *TOPAS* (Coelho, 2007 ▸) controlled by *FIDEL*, and finalized by a user-controlled Rietveld refinement.

### 1.3. Development of the global optimization method

The method for structure determination from unindexed powder patterns described by Habermehl *et al.* (2014 ▸) requires as input either an appropriate structural model or a list of structures, *e.g.* from a crystal structure prediction (CSP). The significant increase in the reliability of CSP (Neumann, 2008 ▸; Reilly *et al.*, 2016 ▸; Neumann & van de Streek, 2018 ▸) is concomitant with a demand for exhorbitant required computing time. When searching for the structure corresponding to just one experimental powder pattern it is simply not necessary to try to find all possible low-energy structures for a given compound. Hence, we transferred the global optimization approach of CSP to the direct fit of crystal structures to powder diffraction data, thus avoiding the major effort for a reliable search for structure candidates by energy minimization. Furthermore, the approach by direct fitting may reveal the existence and kind of disorder in the examined structure, as well as other effects that could be missed by the approach via the screening of CSP results.

We developed a new method for SDPD from scratch by global optimization, *FIDEL-GO* (‘FIt with DEviating Lattice parameters - Global Optimization’), based on the method employed by *FIDEL* for local optimization. Here we describe this global optimization method and its implementation (Section 2). By exploiting the potential and versatility of the pattern comparison approach of *S*_12_, a complete framework for SDPD evolved that comprises almost all scenarios of crystal structure determination from powder data (Section 2.4).

**Figure 1:**
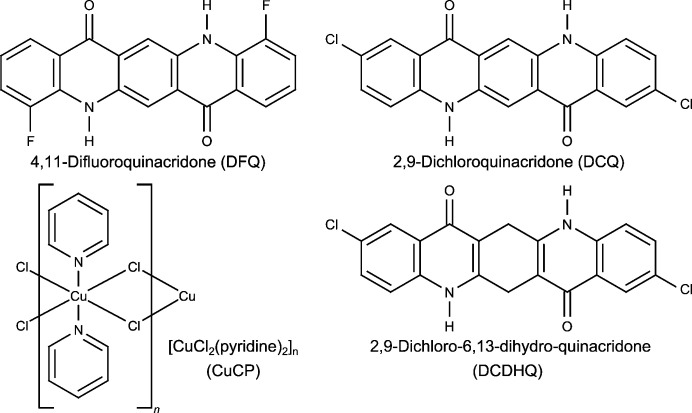
Structural formulae of the application examples.

After giving some computational (Section 3) and experimental (Section 4) details, we present applications (Section 5) of SDPD from scratch with *FIDEL-GO* for four powders (Fig. 1 ▸): (i) the α-phase of 4,11-difluoro-quinacridone (DFQ), (ii) the α-phase of 2,9-dichloro-quinacridone (DCQ), (iii) 2,9-dichloro-6,13-dihydro-quinacridone (DCDHQ) and (iv) CuCl_2_(pyridine)_2_ (CuCP). DFQ, DCQ and DCDHQ are nanocrystalline organic pigments with rigid or semi-rigid molecules. They were chosen to demonstrate how the *FIDEL-GO* method works, and to prove that crystal structures of medium-sized molecules can actually be determined from powder patterns with only about 15 peaks. The limitations of the method are also discussed. The coordination polymer CuCP serves as an example of a moderately flexible compound with ten internal degrees of freedom in the calculation.

## 2. Method

The feasibility and success of SDPD from scratch with *FIDEL-GO* are attributed to the synergy of the following concepts and approaches:

(i) The similarity measure *S*_12_ and the adaptation of its neighbouring range parameter *l* for comparison, fitting and clustering.

(ii) The compact description of structural models with a minimal number of variable parameters.

(iii) The robust and well considered fitting algorithms.

(iv) A suitable setup and handling of the global parameter search space.

(v) The Monte Carlo approach for exploration of the search space.

(vi) The hierarchical search strategy advancing from pre-selection by comparison through several steps of fitting, evaluation and selection of structure candidates to the final refinement.

(vii) A sound overall architecture based on automation, frameworking and interfacing, supported by the integration of many concepts, methods, software and data sources.

**Figure 2:**
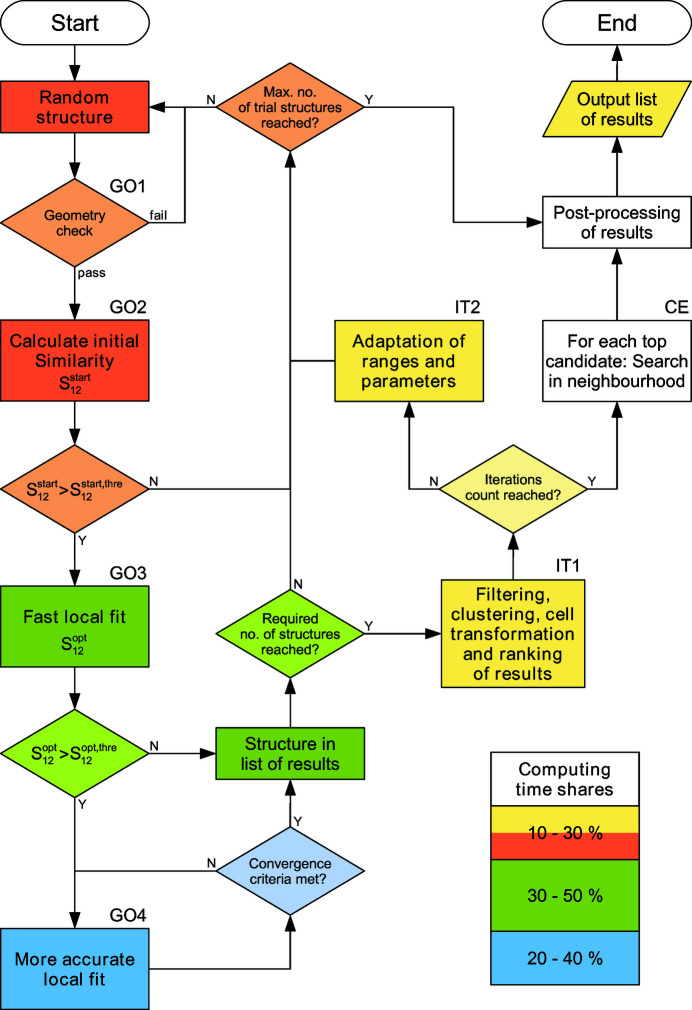
Schematic flowchart of the global optimization run (GO). For details see text (Section 2.3).

The core elements of the new method are the global optimization runs (GO) in selected crystal symmetries (space group, *Z*′, Wyckoff positions). The overall procedure of SDPD from scratch with *FIDEL-GO* consists of the following subsequent stages which will be referred to by their specified acronyms:

(i) GO – global optimization runs (Section 2.3, Fig. 2 ▸), yielding sets of qualified structural models.

(ii) RE – automatic re-evaluation: (*a*) collection, filtering and ranking of the GO results, yielding the primary result set for each crystal symmetry (RE1); (*b*) enhanced *FIDEL* fitting and clustering of structures that reach a high similarity, yielding the final results of the global optimization, a list of top-ranking structure candidates (RE2).

(iii) AR – automatic Rietveld refinement of one or more structure candidates selected by the user based on critical evaluation of the RE2 results.

(iv) DO – geometry optimization by lattice energy minimization of selected structural models using dispersion-corrected density functional theory (DFT-D), if necessary.

(v) UR – user-controlled Rietveld refinement.

### 2.1. Inputs and settings

The following inputs and static settings are required for SDPD from scratch:

(i) A background-corrected powder pattern.

(ii) A molecular geometry model (*e.g.* from geometry optimization).

(iii) Selection of a peak shape function and estimation of FWHM.

(iv) Settings related to instrumentation and measurement parameters.

(v) Selection of internal degrees of freedom.

(vi) Selection of the crystal symmetries and search space setup.

(vii) A 2θ range for the comparison of simulated and experimental patterns (*e.g.* 3–40°).

(viii) An initial neighbouring range parameter *l* (*e.g.* 1–2°).

(ix) Selection and configuration of optimization algorithms and convergence criteria.

In *FIDEL-GO* all of these settings are supported by reasonable defaults or automated procedures for their determination or generation.

### 2.2. Construction of the search space

Each global optimization run is performed in a given crystal symmetry, *i.e.* space group, *Z*′ and the site symmetry of the molecule(s). Likely crystal symmetries for the search can in some cases be derived from indecisive indexing or from the symmetries of related compounds. The general approach to the selection of crystal symmetries for SDPD from scratch, however, is based on the space group statistics of the Cambridge Structural Database (CSD; Groom *et al.*, 2016 ▸). The statistical analysis by Pidcock *et al.* (2003 ▸) is used to identify the most common crystal symmetries for the mol­ecular symmetries. The selection of space groups and special positions is usually fine-tuned based on crystallographic experience.

For every fitted parameter and for the cell volume sensible ranges are defined. Minimum and maximum values of the unit-cell parameters are derived from the spatial dimensions of the molecules. The ranges for the parameters describing the position and orientation of the molecules are set according to the characteristics of the space group and the site symmetry. The ranges for conformational degrees of freedom are set considering chemical plausibility and molecular symmetry. The range for the cell volume is set according to the estimated molar volume based on volume increments given by Hofmann (2002 ▸) or to known crystal densities of related phases or compounds. The parameter and volume range settings apply only to the starting structure and do not constrain the trajectories of local optimization runs. The local optimization with the preferred hill-climb algorithm, however, can include restraints on the fitted parameters when they approach the range boundaries.

### 2.3. Global optimization

The general problem of global optimization approaches to SDPD lies in the huge amount of computing time required, even if the number of fitted parameters is comparatively small. Powder patterns are highly sensitive to small changes in the crystal structure. This is an essential advantage for SDPD, but generates a major obstacle to SDPD from scratch without prior indexing. In structure fitting to powder data the most time-consuming computational task is the simulation of powder patterns from the structural models. The characteristics of the similarity measure *S*_12_ are very well suited to coping with this problem. *S*_12_ facilitates the detection of a rough match of a trial structure to the powder data using a broad weighting function *w*(*r*). This allows for an effective pre-selection of suitable trial structures by comparison. Furthermore, the similarity hypersurface is smoothed out by the use of a relatively broad weighting function, which is in favour of fast local optimizations going in the right direction. Successive narrowing of the neighbouring range leads to a more accurate fit. The combined approach of pre-selection and local optimizations under the general regime of successively reducing an initially broad neighbouring range of *S*_12_ is the major key to the general applicability, scalability, efficiency and effectiveness of the method. It allows for a drastic reduction in the number of time-consuming pattern simulations, while still being very specific in the search for the best match. The global optimization method of *FIDEL-GO* takes into account these characteristics of the problem and of *S*_12_ by employing a complex hierarchical search strategy.

The procedure of a global optimization run in a given crystal symmetry is summarized in Fig. 2 ▸. The GO run is based on the generation of random trial structures. Each random structure passes through up to four successive steps with increasing computing effort:

(i) GO1 – a check of cell volume and geometry (rejection of structures with too close contacts of atoms).

(ii) GO2 – pre-selection of trial structures by comparison of the simulated and experimental patterns using a broad weighting function, yielding 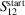 .

(iii) GO3 – a fast local fit: raw structure fitting with a fast (usually conjugated gradient) optimization algorithm, yielding 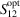 .

(iv) GO4 – a cycle of more accurate local fits with a better but more time-consuming hill-climb algorithm, a narrowing weighting function and successively stricter convergence criteria.

The conditional steps GO3 and GO4 are triggered by the two threshold levels 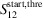 and 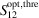 , respectively, that are adjusted dynamically during the GO run so that the relative computing times for the generation and pre-selection of trial structures (GO1–GO2), the raw structure fitting (GO3) and the post-optimization cycle of more accurate fittings (GO4) are balanced. This approach ensures that enough random structures are evaluated and allows the procedure to adapt automatically to various conditions resulting from the crystal symmetry and the experimental powder data.

In the initial setup the search space is huge with respect to the unit-cell parameters. While the cell volume constraint already cuts out only a small part, the search space for the unit-cell parameters is still highly redundant in terms of crystallographic equivalence. The qualified structures accumulating in the list of results all come from the local optimization (GO3–GO4) with a wide convergence radius, in particular regarding the response of the unit-cell parameters to the dominant signals in the observed pattern. Accordingly, at least the best models are found multiple times and the result set is subjected to clustering of similar structures. Moreover, the unit-cell parameter search space is populated with qualified candidates rather selectively during the global search. These structural models appear with different unit-cell settings. After the automatic cell transformation, at least some of the unit-cell parameters usually show a monomodal distribution, thus effectively implying a fuzzy indexing of the powder data. The fitted parameters of the molecules (*m_i_*, φ_*i*_, τ_*i*_) may exhibit a more or less pronounced mono- or multimodal distribution pattern as well, *e.g.* due to the steric hindrance of torsions or the stacking of planar molecules. This valuable information evolving during the global search is exploited by an auto-focusing mechanism that dynamically adapts the search space based on the population analysis of structures with high 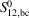 .

The overall procedure passes through a number of iteration steps, where

(i) The list of qualified structural models is subjected to filtering, clustering and subsequent automatic cell transformation (labelled IT1 in Fig. 2 ▸),

(ii) The search space is narrowed based on the statistical evaluation of the structure candidates found thus far (IT2),

(iii) The weighting function for the similarity computation is narrowed (IT2), and

(iv) The convergence criteria become stricter (IT2).

The search ranges are carefully narrowed, reacting primarily to pronounced monomodal parameter distributions, in particular with respect to the unit-cell parameters. Since the trajectory of local structure fits is allowed to go beyond the borders of the actual search space, the automatic adaptation can even shift or widen the search ranges. A similar approach of using dynamic boundaries driven by the evolving parameter distributions is also used in *e.g.* SDPD by the evolutionary direct-space method of Chong & Tremayne (2006 ▸).

The filtering out and rejection of unsuitable structures and the evaluation of potential structure candidates is primarily based on their reference similarity 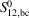 (Section 1.1). Each candidate is also characterized by the weight *W*_C_, an integer value indicating the number of levels (GO1–GO3 and cycles of GO4) it has passed. The cell volume, and optionally the result of a single-point force-field energy calculation *E*_FF_, are used as additional descriptors.

The clustering of structure candidates is based on the pairwise comparison of simulated powder patterns using *S*_12_ with a narrow neighbouring range and a high similarity threshold for the grouping of structures. Every cluster is represented by the structure that exhibits the highest 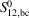 . The other structures of the cluster are discarded and the value of their weight descriptor *W*_C_ is added to the *W*_C_ of the top candidate that represents the group. Of course, all structure candidates in the list of results fit the observed pattern to a considerable extent, which is typical of low-quality experimental data. The simulated powder patterns used for structure comparison are much more detailed and a large number of medium and weak reflections allows the differentiation of structures that are actually different. Any clustering runs the risk of concealing significant differences, thus merging structures incorrectly if the criteria are too tolerant. This is also true for existing polymorphs compared via the *S*_12_ of simulated patterns (Sacchi *et al.*, 2020 ▸). Optionally, *FIDEL-GO* can use a more secure variant that tests the structural similarity by fitting the lower-ranking structural model to the simulated pattern of the top candidate. If the simulated patterns become identical, the structures are indeed duplicates. However, this is very time-consuming and normally not necessary. The clustering is always carried out with sufficiently strict criteria, thus leading to a certain persistence of structure candidates that turn out to be equivalent at a later stage.

After the general exploration of the search space the best ranking structural models can be re-evaluated by targeting the global optimization procedure at parameter search regions in their neighbourhood (labelled CE in Fig. 2 ▸). While being a Monte Carlo method in the first place, the global optimization method of *FIDEL-GO* also includes mechanisms corresponding to other optimization approaches. The hierarchy of conditional random structure evaluations and fits (GO2–GO4) is similar to simulated annealing approaches. The iterative adaptation of parameter ranges (IT2) and the re-evaluation of search-space regions in the neighbourhood of top structure candidates (CE) resemble characteristics of evolutionary algorithms.

### 2.4. SDPD procedure and application framework

The result sets of one or more global optimization runs (GO) in each of the selected crystal symmetries are collected, filtered and ranked, yielding the primary result set for each crystal symmetry (RE1). The standard filter criteria account for the agreement with the powder data 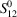 , a sensible molar volume and a maximum number of candidates considered. Subsequently, the top ranking structures of the primary results are subjected to an automatic re-evaluation procedure including enhanced fitting and clustering (RE2), yielding the final results of the global optimization by *FIDEL-GO*. The enhanced fitting is performed using a larger 2θ range, smaller *l* values (0.1–0.2°) and stricter convergence criteria. Furthermore, the fine fit may include an improved profile modelling or the fitting of additional internal degrees of freedom.

After the evaluation of similarity values, molar volumes and crystal structures, and the visual comparison of the simulated powder patterns with the experimental data by the user, the SDPD procedure succeeds with automatic Rietveld refinements (AR) of selected promising structures. The structure determination is finalized by a careful and sound user-controlled Rietveld refinement (UR).

DFT-D geometry optimizations (DO) of selected structures can be employed in order to gain valuable hints in the case of persistent ambiguities of different structural models. In particular, in the case of ‘problematic’ powder data the global optimization may provide several different models that are chemically sensible and match the experimental data similarly well. Crystal structures can be validated by lattice energy minimization using DFT-D, as has been shown by Neumann and van de Streek (Neumann *et al.*, 2008 ▸; van de Streek & Neumann, 2010 ▸; van de Streek & Neumann, 2014 ▸).

**Figure 3:**
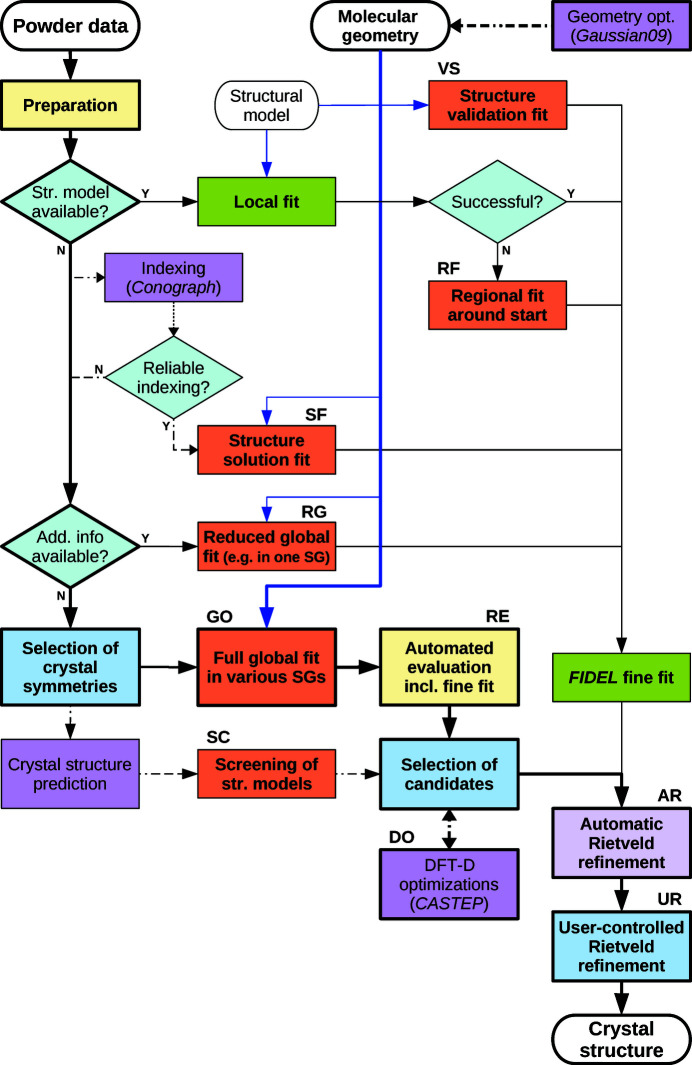
Schematic flowchart describing the application framework for SDPD with *FIDEL-GO*. The general overall procedure for SDPD from scratch as described in this work is indicated by bold lines and borders. Applications of the global optimization method are shown in orange. Local structure fitting is shown in green. Major decisions and actions by the user are depicted in blue. The integration of optional third-party external programs is indicated by purple boxes and dashed paths. Yellow refers to stages inheriting multiple tasks that are largely automated but need some user interaction.

The overall SDPD procedure is outlined in Fig. 3 ▸. The global optimization method is primarily targeted at SDPD from scratch. However, *FIDEL-GO* has evolved into an almost comprehensive application framework suitable for a wide range of application scenarios. By specific configuration of the global optimization runs, the method can easily be adapted to a variety of ‘less global’ applications, described below. The flowchart in Fig. 3 ▸ also shows how several auxiliary third-party components and specific adaptations of the global optimization runs fit into the hierarchy of procedures that make up the general framework.

#### 2.4.1. Structure solution fit (SF)

If the unit-cell parameters are known from indexing or from isostructural compounds, the structure solution is carried out with very narrow ranges for the unit-cell parameters. The use of narrow ranges instead of fixed unit-cell parameters takes into account the limited accuracy of the indexing results and adds some flexibility to local optimization trajectories.

#### 2.4.2. Reduced global fit (RG)

If the indexing of a powder pattern is substantially uncertain or incomplete, the procedure can be run in one or a few space groups using specific range settings for the unit-cell parameters. This is a valuable approach, *e.g.* for patterns which are dominated by *hk*0 reflections, so that a*, b* and γ* can easily be determined, whereas information on c*, α* and β* is low or even completely absent.

#### 2.4.3. Regional fit (RF)

The search space can be automatically centred around a given starting structure using comparatively small parameter ranges, *e.g.* whenever a local *FIDEL* fit as described by Habermehl *et al.* (2014 ▸) cannot successfully capture the structure, or when the fitting to the observed pattern leads to an obviously wrong local similarity maximum. The regional fit can also be used for the validation of a structure solution (VS), *i.e.* to check whether a structural model is really the best match to the powder data within a narrow parameter hyperspace region (see Gorelik *et al.*, 2021 ▸).

#### 2.4.4. Screening of large sets of structural models (SC)

The random trial structures that are usually the starting point of the global optimization can be replaced by a list of input structures, *e.g.* from a CSP (see Section 5.2.2) or sets of possibly isotypical structures of chemical derivatives, solvates or hydrates. Thus the procedure turns into a tool for the automated screening, fitting, clustering and ranking of structure candidates for an experimental pattern.

## 3. Computational details

*FIDEL-GO*, the global optimization approach to SDPD described above, has been implemented by extension of the program *FIDEL* (Habermehl *et al.*, 2014 ▸). The program is essentially a highly configurable non-interactive command-line tool supporting simple *ad hoc* calls as well as complex workflows. The core executable is written in ISO C with bindings to C and C++ libraries. It supports parallel execution of time-consuming tasks on multi-core machines. Active development and application is done on various Windows and Linux systems. The program supports the construction of complex process chains using *FIDEL-GO* features, XSL transformations (see Section 3.2), template-based outputs and the integration of external programs. A menu-based user interface allows fast interactive work with *FIDEL*. The *FIDEL-GO* calculations require a computational effort comparable to that of a CSP with force fields. A typical full global optimization run in one space group with 10^6^ random trial structures took about 12 hours (range 3–30 hours) on an Intel Core i7 at 3.2 GHz. At present *FIDEL-GO* can only be used by an experienced user, therefore it is not yet included in the commercial version of the program *FIDEL*.

### 3.1. Automation and performance

Automation and performance are two crucial aspects regarding the feasibility of approaches to SDPD for practical applications. Hence, substantial effort was made to meet these requirements by appropriate architectures for the methods and computational procedures. At the methodological level, both requirements are met by employing a flexible hierarchy of scalable or adaptive procedures. At the implementation level, a key to automation was the integration of existing methods, functions, programs and software libraries, in particular by integrating established or innovative open source software in the fields of chemistry, crystallography and diffraction. At the computational level, performance is supported by parallelization of major time-consuming computations on multi-processor machines. Performance options at different levels of the general procedure include the caching of reflection lists, intensity corrections and scattering factors, neglecting of hydrogen atoms, and the limitation of the 2θ range for powder pattern simulation. The general performance perspective of distributed computing is implicitly supported by any global optimization approach and explicitly supported by *FIDEL-GO* and its procedures.

Automation features include:

(i) Background correction based on the Bayesian algorithm provided by *ObjCryst++* (Favre-Nicolin & Černý, 2002 ▸).

(ii) Determination of an appropriate peak shape function and estimation of FWHM based on analysis of the diffractogram and peak extraction by *PeakSearch* (Oishi-Tomiyasu, 2012 ▸), followed by optimization of the FWHM value by *FIDEL-GO*. The optimization is done by fitting a powder pattern simulated from the extracted peak positions and intensities to the experimental data using *S*_12_ with a small *l* value. This does not include indexing attempts nor the use of a structural model.

(iii) Construction of a *z*-matrix representation of the molecule(s) that allows a reasonable fit of molecular arrangements and flexibilities (see Section 1.2). Degrees of freedom for rotatable bonds and for the movement of fragments can be determined automatically using the structure analysis features of the *OpenBabel* library (O’Boyle *et al.*, 2011 ▸).

(iv) Determination of constraints for the structure fitting due to space group and site symmetry, supported by *cctbx* (Grosse-Kunstleve *et al.*, 2002 ▸).

(v) Determination of the expected cell volume range based on volume increments according to Hofmann (2002 ▸).

(vi) Clustering, ranking and filtering of sets of structural models using *S*_12_, complemented by evaluation of other descriptors such as molar volumes and lattice energies calculated by integrated force-field routines from *CRYSCA* (Schmidt & Englert, 1996 ▸; Schmidt & Kalkhof, 1998 ▸).

(vii) Generation of restraints for the Rietveld refinements based on CSD statistics provided by *Mogul* (Bruno *et al.*, 2004 ▸).

(viii) Automatic Pawley fit and Rietveld refinement sequence with *TOPAS* (Coelho, 2007 ▸) called by *FIDEL*.

Other third-party software programs used to facilitate certain tasks and to complement existing features of *FIDEL-GO* are: *Conograph* (Oishi-Tomiyasu, 2014 ▸; Esmaeili *et al.*, 2017 ▸) for attempts to index the powder data, *GAUSSIAN09* (Frisch *et al.*, 2009 ▸) for optimization of the molecular geometry, *CASTEP* (Clark *et al.*, 2005 ▸) for the validation of crystal structures, *PLATON* (Spek, 2009 ▸) for structure analysis and a search for higher symmetry, *Gabedit* (Allouche, 2011 ▸) for *z*-matrix visualization and editing, *GSL* (Galassi *et al.*, 2009 ▸) and *R* (R Core Team, 2017 ▸) for the statistical analysis of the parameter search space and the similarity hypersurface, *gnuplot* (Williams & Kelley, 2014 ▸) for data and diagram visualization, and *Jmol* (Hanson, 2010 ▸) for interactive crystal structure visualization on the report pages.

### 3.2. Interfacing, data processing, reporting and visualization using XML technologies

The development of complex scientific software in the academic domain faces some major problems with regard to usability and flexibility of the software in practice, as well as perspectives for long-term development. In addition to the requirements in terms of automation and performance, it is important to provide the user with suitable means for (i) preparing, adapting and configuring the inputs, (ii) customizing project design and control, (iii) easy evaluation, visualization and further processing of the results, and (iv) interfacing with other programs. Besides the implementation of basic capabilities to define and execute complex process chains, these objectives have primarily been achieved by the use of open standards and technologies based on XML (extensible markup language):

(i) XML and CML (chemical markup language) (Murray-Rust & Rzepa, 2011 ▸) for input and output of configurations, results and chemical structures.

(ii) Interactive HTML pages and SVG graphics for reporting, visualization and publishing.

(iii) XSLT (extensible stylesheet language transformation) for data processing, interfacing and report generation.

Thus a high degree of flexibility, customizability, transparency, validatability and portability can be realized while minimizing the non-trivial installation prerequisites and requirements regarding the programming skills of advanced users.

## 4. Experimental details

### 4.1. X-ray powder diffraction

X-ray powder diffraction (XRPD) data of the samples of DFQ, DCQ, DCDHQ and CuCP (Fig. 1 ▸) were recorded with Cu *K*α_1_ radiation in transmission mode at room temperature. The samples were measured between polymer films on a Stoe Stadi-P diffractometer equipped with a curved Ge(111) primary monochromator and a linear position-sensitive detector. The program suite *WinX^POW^* (Stoe & Cie, 2006 ▸) was used for data collection. Details are provided in Section S1 in the supporting information.

### 4.2. Quantum-mechanical calculations

The initial molecular geometries of DFQ, DCQ and DCDHQ were obtained by geometry optimization using *GAUSSIAN09* (Frisch *et al.*, 2009 ▸), DFQ and DCDHQ at the HF/6-31G** level, and DCQ at the MP2/6-31G* level.

Selected crystal structure models were subjected to DFT-D lattice energy minimization (Neumann, 2008 ▸; van de Streek & Neumann, 2010 ▸) with *CASTEP* (Clark *et al.*, 2005 ▸), using the combination of the PBE functional (Perdew *et al.*, 1996 ▸) and the semi-empirical dispersion correction according to Grimme (2006 ▸). The convergence criteria were set to ‘fine’ level (energy tolerance 10^−5^ eV atom^−1^, maximum force tolerance 0.03 eV Å^−1^, maximum stress tolerance 0.05 GPa, maximum displacement tolerance 0.001 Å). At first the energy minimizations were performed with fixed unit-cell parameters to yield improved structural models and molecular geometry restraints for the Rietveld refinement. For further validation and energy ranking the resulting structural models were then optimized without constraining the cell dimensions.

### 4.3. Crystal structure prediction

For DCQ a crystal structure prediction was performed in the most common space groups using the force-field program *CRYSCA* (Schmidt & Englert, 1996 ▸; Schmidt & Kalkhof, 1998 ▸). *CRYSCA* performs a global lattice energy minimization, starting from a set of 10^5^–10^7^ random structures. The optimized structures are ranked by energy. The structure prediction is continued until the lowest-energy structures have been found several times from different starting points. In *CRYSCA* a crystal structure is described in the same way as in *FIDEL-GO* (see Section 1.2). The intermolecular interactions were treated as a sum of van der Waals, hydrogen-bonding and Coulomb terms. For the van der Waals potentials the DREIDING parametrization in its recommended 6-exp form (Mayo *et al.*, 1990 ▸) was used. Hydrogen-bond energies were calculated using a self-developed 10–12 potential without angle dependency, but this potential did not yield very accurate structures. The Coulomb energy was calculated from atomic charges derived by the electrostatic potential (ESP) approach (Chirlian & Francl, 1987 ▸).

### 4.4. Rietveld refinements

All Rietveld refinements were performed using *TOPAS Academic*, Versions 4.1, 4.2 and 6 (Coelho, 2007 ▸, 2009 ▸, 2016 ▸, 2018 ▸). The robust automatic refinement procedure in stage AR consists of a sequence of seven *TOPAS* calls configured and controlled by *FIDEL*. Automatic refinements of structural models with *Z*′ < 1 were performed in subgroups with *Z*′ ≥ 1. Restraints for bond lengths and bond angles were usually automatically derived from median values from CSD statistics provided by *Mogul* (Bruno *et al.*, 2004 ▸). Additional ‘flatten’ restraints were applied for planar moieties. The final user-controlled Rietveld refinements in stage UR usually started from the structural models obtained by the automatic Rietveld refinements. Molecular geometry restraints for the user-controlled refinements were preferably taken from DFT-D calculations. Details appear in Section S2 in the supporting information.

## 5. Applications

### 5.1. SDPD of DFQ by global optimization

**Figure 4:**
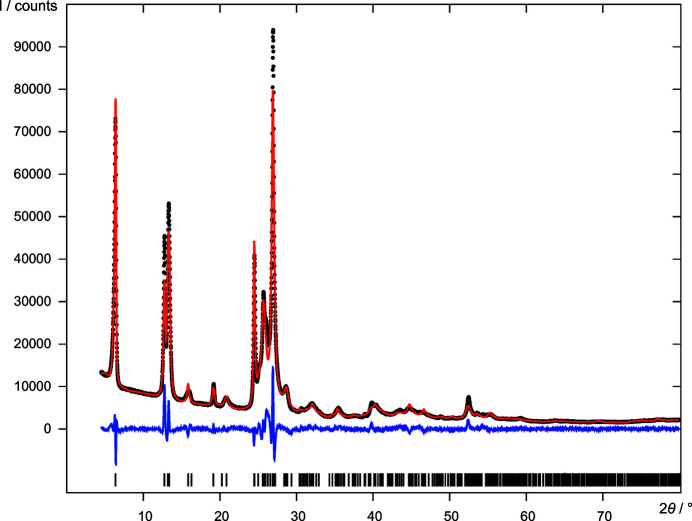
Final Rietveld refinement of DFQ (*P*2_1_/*c*, *Z*′ = 0.5, model *B*), showing the experimental X-ray powder diagram (black dots), the simulated diagram of the refined structure (red line), the difference curve (blue line) and reflection positions (black bars).

**Figure 5:**
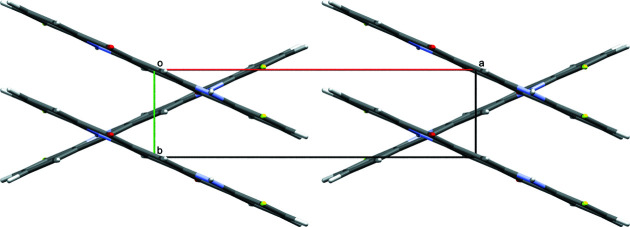
The crystal structure of DFQ (*P*2_1_/*c*, *Z*′ = 0.5, model *B*). The view is along [001].

4,11-Difluoro-quinacridone (C_20_H_10_F_2_N_2_O_2_, DFQ, Fig. 1 ▸) is a non-commercial orange pigment. The corresponding chloro derivative 4,11-dichloro-quinacridone is polymorphic with four described polymorphs (Hunger & Schmidt, 2018 ▸). Its β-phase crystallizes in *Pbca*, *Z* = 4 (Chung & Scott, 1971 ▸). For DFQ no crystal structures are known. The powder pattern of DFQ exhibits only about nine sharp reflections and a number of broad humps (see Fig. 4 ▸). Reliable indexing is not possible. Nevertheless, this powder pattern was sufficient to determine the crystal structure (Fig. 5 ▸) using the global optimization method of *FIDEL-GO*.

In the SDPD procedure the molecule was treated as a rigid body of the point group *C*_2h_. According to space-group statistics, about 95% of all molecules with *C*_2h_ symmetry are located on crystallographic inversion centres (Pidcock *et al.*, 2003 ▸). Hence, global optimization runs were performed in the space groups 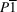 , *P*2_1_/*c*, *Pbca* and *C*2/*c* with molecules on inversion centres, and additionally in statistically common space groups with molecules on the general position (*P*2_1_, *P*2_1_/*c*, *P*2_1_2_1_2_1_), and in *P*1, *Z* = 1. Additional runs were performed in *C*2/*m* with molecules on positions with site symmetry 2/*m* (see Table 1 ▸). The molar volume of quin­acridone derivatives is usually about 10% smaller than the predicted volumes based on the volume increments of Hofmann (2002 ▸), due to the dense π–π stacking of the aromatic systems. Hence, the volume range *V*/*Z* for the global optimization of DFQ was set to roughly 365 Å^3^ ± 10%.

The stepwise evolution of the SDPD process is outlined in Table 1 ▸. After initial checks of volume and geometry (GO1) a total number of about 21 000 000 trial structures were evaluated by simple comparison of the simulated and observed powder patterns (GO2). Only ∼137 000 of the trial structures (0.65%) qualified and were fitted to the experimental pattern (GO3). Of these fitted structure candidates, 7828 (5.7%) also entered the post-optimization cycle of more accurate fits (GO4). An overview of the primary result structures with the highest 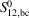 values in each of the nine crystal symmetries is shown in Table 2 ▸.

The re-evaluation fine fit led to a list of 122 structural models (RE2). The best 61 of them with reference similarities 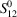 between ∼0.98 and 0.99 in effectively three crystal symmetries were inspected as candidates for selection by the user [see Table 3 ▸(*a*), and Table S2 in the supporting information]. Eighteen structure candidates were selected for the evaluation by automatic Rietveld refinements (AR), resulting in *R*_wp_ values of 7–17% [Table 3 ▸(*b*)].

A unique solution, which is better than all the others, could not be identified. Four structural models gave a similarly good fit to the experimental data and exhibited chemically reasonable packing motifs, each of them found several times with minor differences. However, the packing in each instance is considerably different:

Model *B*: *P*2_1_/*c* (*Z*′ = 0.5). Criss-cross pattern similar to the γ-phase of unsubstituted quinacridone (Paulus *et al.*, 2007 ▸) (*cf*. Fig. 5 ▸).

Model *A*: *P*2_1_/*c* (*Z*′ = 1). Criss-cross packing motif similar to model *B*, but in a cell double the size of model *B*.

Model *C*: 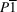 (*Z*′ = 0.5). Chains forming a layered structure similar to DCQ (Section 5.2) and the α^I^-phase of unsubstituted quinacridone (Paulus *et al.*, 2007 ▸).

Model *D*: *P*2_1_/*c* (*Z*′ = 1). A combination of the packing motifs of models *B* and *C* that has so far not been found in any phase of quinacridone or its derivatives.

DFT-D calculations of structural models *A*–*D* with *CASTEP* (DO) confirmed the close correspondence of models *A* and *B* and revealed a significantly lower likelihood of models *C* and *D* [Table 3 ▸(*c*)].

Finally, six candidates were subjected to a user-controlled Rietveld refinement (UR). Two pairs turned out to be duplicates, leaving four different structures (*A*, *B*, *C* and *D*) [Table 3 ▸(*d*)]. The top ranking structure *A* led to a better Rietveld fit to the data than *B*, but at the cost of double the number of atom position parameters. Structures *A* and *B* are virtually identical. In *A* the molecules are located on a general position in *P*2_1_/*c*, *Z* = 4, while in *B* they are located on an inversion centre in *P*2_1_/*c*, *Z* = 2. Bearing in mind the limited quality of the diffraction data, the deviation from the higher symmetry in structure *A* is not significant. Hence, model *B* (Fig. 5 ▸, CIF file in the supporting information) having the higher symmetry should be regarded as the correct one. Its final Rietveld plot is shown in Fig. 4 ▸. The hypothesis that *B* is the correct structure was later confirmed by further investigations, including solid-state NMR measurements, alternative DFT-D calculations and fits to the pair distribution function (Schlesinger *et al.*, 2022 ▸).

### 5.2. SDPD of DCQ by global optimization or by screening of CSP results

**Figure 6:**
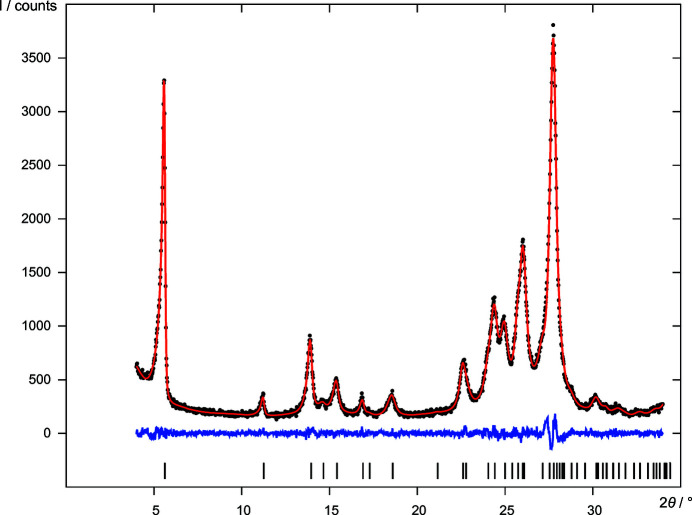
DCQ: final Rietveld refinement in 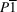 , *Z* = 1. For the diagram legend see Fig. 4 ▸.

2,9-Dichloro-quinacridone (C_20_H_10_Cl_2_N_2_O_2_, DCQ, Fig. 1 ▸) is an industrial red pigment used for automotive coatings (Hunger & Schmidt, 2018 ▸). The structures of the γ- and δ-phases, which are formed by high-temperature recrystallization or sublimation, are known from single-crystal X-ray analyses (Senju *et al.*, 2005*a* ▸,*b* ▸). The crystal structure of the α-phase, which is formed during the synthesis, is hitherto not known. The α-phase is inherently nanocrystalline (see Fig. 6 ▸). The pigment is nearly insoluble in all solvents, even at elevated temperatures. All recrystallization attempts failed. It was not even possible to improve the crystallinity: under mild conditions the powder pattern of the sample did not improve, while under harsher conditions the phase changed to the more stable γ-phase.

The XRPD pattern of the α-phase contains only about 14 reflections and cannot be indexed reliably. Here we present the structure determination of the α-phase using *FIDEL-GO* in two different ways: (i) by structure solution from scratch with a global *FIDEL-GO* fit and (ii) using *FIDEL-GO* to screen the results of CSP (see Section 4.3).

#### 5.2.1. SDPD from scratch by global optimization

The global optimization runs were performed in a similar way to that described for DFQ (see Section 5.1). The best primary result structures in each of nine crystal symmetries (RE1) are shown in Table S6 in the supporting information. The final results of the global optimization (RE2) yielded 60 structure candidates with 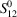 values between 0.97 and 0.99 (see Table 4 ▸). The best ranking structure solution with a reference similarity 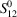 of 0.987 was found in *P*1 (*Z*′ = 1) and in 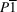 (*Z*′ = 0.5). The second best model, also in 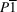 (*Z*′ = 0.5), had an 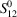 value of 0.981, but an unrealistically low molar volume.

**Figure 7:**
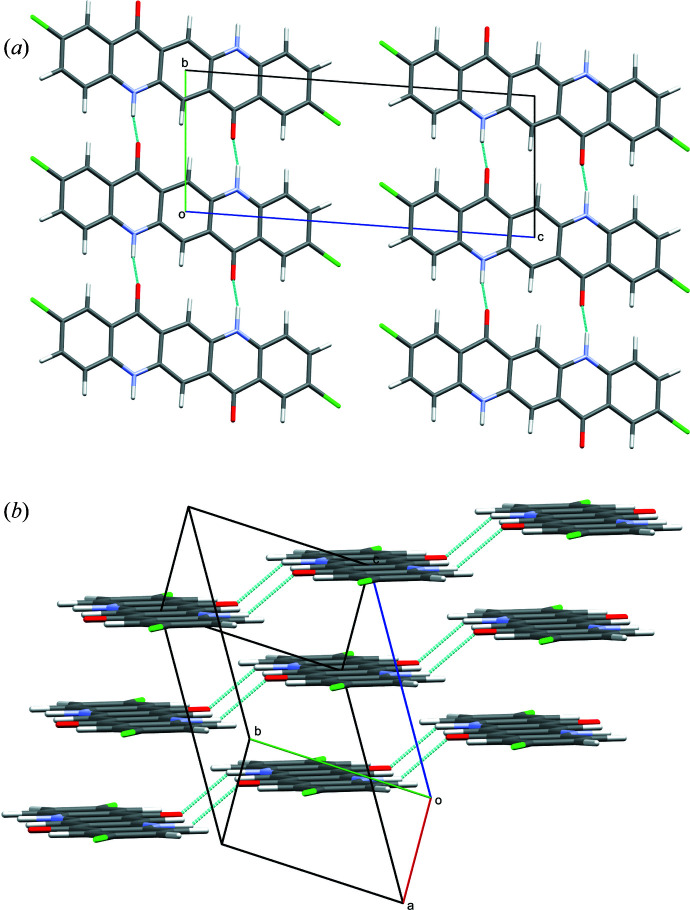
The crystal structure of the α-phase of DCQ ( 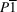 , *Z* = 1): (*a*) viewed along [100] and (*b*) viewed perpendicular to the chains.

The evolution of the SDPD of DCQ from the initial trial structure to the final Rietveld refinement is presented in Table 5 ▸ for the example of the candidate that led to the correct structure. The best structural model in 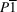 , *Z* = 1 from the global optimization with *FIDEL-GO* showed a reasonable molar volume and packing, and matched the experimental data significantly better than all the other candidates. The DFT-D geometry optimization (DO) of this structure indicates its correctness too. The structure changed only slightly during geometry optimization with fixed unit-cell parameters (see Fig. S1 in the supporting information). The final user-controlled Rietveld refinement (UR) resulted in a good fit to the powder data (Fig. 6 ▸). The structure is shown in Fig. 7 ▸ and available in the CIF file in the supporting information. The molecules are arranged in chains parallel to the [110] direction. Each molecule is connected to two neighbouring mol­ecules via double hydrogen bonds [Fig. 7 ▸(*a*)]. The chains are not fully planar but exhibit steps of 1.4 Å between neighbouring molecules [Fig. 7 ▸(*b*)]. The final crystal data are given in the supporting information.

#### 5.2.2. SDPD by screening of CSP results

**Figure 8:**
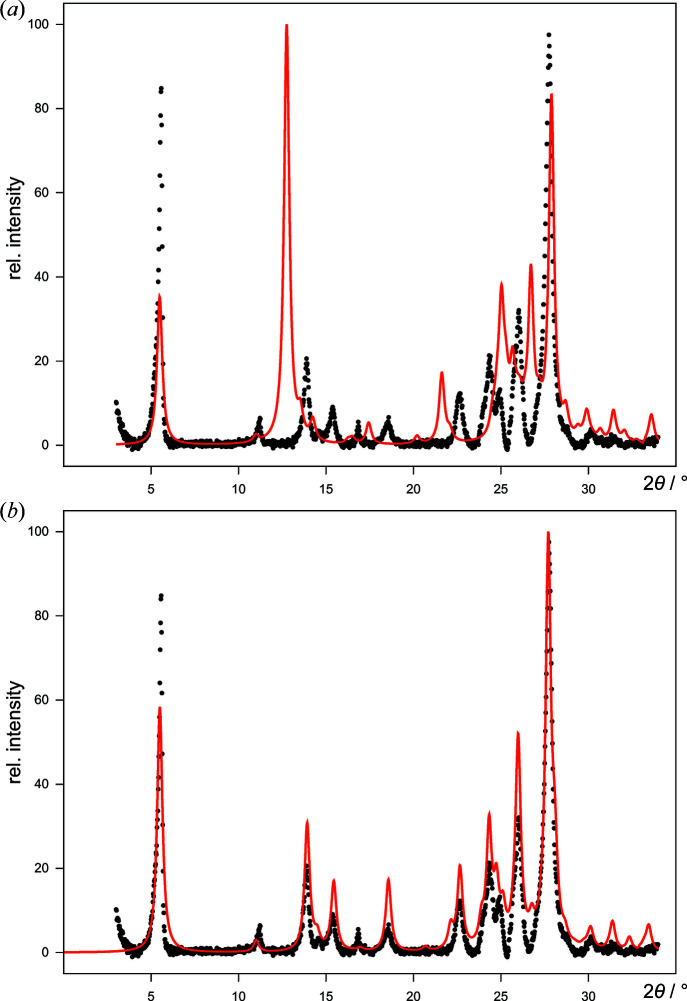
The best structure of DCQ in 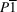 , *Z* = 1 from the screening of the CSP results, showing the background-corrected experimental powder pattern (dots) versus simulated patterns (red lines) of the structural model (*a*) before and (*b*) after the *FIDEL* fit.

Possible crystal structures of DCQ were predicted by global lattice energy minimizations using the program *CRYSCA* (Section 4.3). An overview of the CSP results is shown in Table S7 in the supporting information. None of the predicted structures showed a powder diagram similar to the experimental one. A total of 2190 low-energy structures from CSP in different crystal symmetries were screened by local fitting to the powder data with *FIDEL-GO* (Table 6 ▸). The highest reference similarity 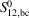 was obtained for a structure candidate in *P*1, *Z* = 1 [Fig. 8 ▸(*a*)]. After the *FIDEL* fit the resulting simulated powder pattern of this structure was quite similar to the experimental one [Fig. 8 ▸(*b*)]. The unit-cell parameters changed by up to approximately 0.6 Å or 1°, while the molecular orientation changed by up to ∼3° during the fit. The example in Fig. 8 ▸ impressively demonstrates the power of a local fit with *FIDEL*. The resulting structure has 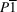 , *Z*′ = 0.5 symmetry and is practically identical to the structure determined by global optimization from scratch (Section 5.2.1).

### 5.3. SDPD of DCDHQ by global optimization

**Figure 9:**
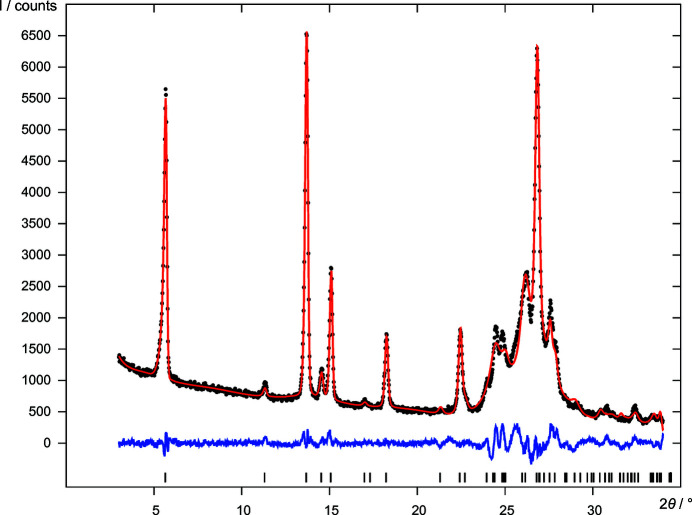
DCDHQ: user-controlled Rietveld refinement of the structural model *B*1*Z*1 in 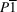 , *Z* = 1. For the diagram legend see Fig. 4 ▸.

2,9-Dichloro-6,13-dihydro-quinacridone (C_20_H_12_Cl_2_N_2_O_2_, DCDHQ, Fig. 1 ▸) was chosen as a first example of a molecule with a certain degree of conformational flexibility. The compound is a precursor in the industrial synthesis of DCQ (Hunger & Schmidt, 2018 ▸). Like DCQ, DCDHQ is obtained as a poorly crystalline powder, with an X-ray powder diagram that contains only about 17 reflections and cannot be reliably indexed (see Fig. 9 ▸). Because of the two *sp* ^3^ carbon atoms in the central ring the molecule shows some conformational flexibility. According to the CSD, the majority of similar molecules show a tilted conformation (for details see Section S5.1 in the supporting information). For DCDHQ, two intramolecular degrees of freedom were considered, allowing for a twist and a tilt of the central ring. Global optimization runs with the two intramolecular degrees of freedom were performed in space groups 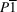 , *P*2_1_, *C*2/*c*, *P*2_1_/*c*, *P*2_1_2_1_2_1_, *Pbca* and *Pna*2_1_ with the molecule on the general position. Additional runs were performed in space groups 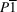 and *P*2_1_/*c* with a rigid planar molecule on an inversion centre (see Table S8 in the supporting information).

The best primary result structures (stage RE1) in each of the nine crystal symmetries are listed in Table S9 in the supporting information. The 400 structural models with the highest similarity values were re-evaluated by a fine fit (stage RE2), yielding 99 structures in five different crystal symmetries with reference similarities 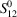 between 0.98 and 0.99, and *R*_wp_ values in the range of 15–24% (Table 7 ▸). The comparatively high *R*_wp_ values are caused by the simple powder pattern simulation of *FIDEL-GO* (static background correction, simple reflection profile, constant FWHM; see Section 1.2). Such a simple pattern simulation would not be suitable in a Rietveld refinement, but is fully sufficient for evaluation of the similarity measure *S*_12_, which depends much less on good modelling of the powder pattern concerning peak profiles, background, intensity corrections *etc*. Table 7 ▸ reveals that there are many structures that have similar unit-cell parameters (4, 6–7 or 16 Å, or doubled values) despite having different space groups and *Z* values. Apparently, these values reflect the major peak positions in the powder pattern. A similar situation is frequently observed for unsatisfactory indexing attempts of the powder pattern of a poorly crystalline sample.

After thorough evaluation by the user, 16 structures were selected for the automatic Rietveld refinement (stage AR, Table S10 in the supporting information) and eight of them were subjected to DFT-D geometry optimization (stage DO, Table S11 in the supporting information). In the automatic Rietveld refinements the *R*_wp_ values dropped to roughly half of the values already achieved by *FIDEL-GO*, due to better modelling of peak profiles and background, and refinement of all atomic coordinates.

Finally, five structures were subjected to user-controlled Rietveld refinements (stage UR, Table 8 ▸). The two structural models *A*3 and *B*1 exhibited the lowest energy in the DFT-D geometry optimization with free unit-cell parameters. Since their unit-cell parameters had changed substantially in the DFT-D calculations, the local fitting procedure of *FIDEL* was applied to re-adjust the unit-cell parameters of these models to the powder pattern before the final Rietveld refinement. The crystal data for models *B*1*Z*1, *B*1, *C*1 and *A*3 from the Rietveld refinement and the DFT-D optimized structure of *A*3 are available in the supporting information.

The powder pattern is dominated by 0*kl* reflections (*h*0*l* for model *C*1). All information about a*, β* and γ* is buried in the broad group of peaks at 24–28°. Hence, different unit cells match the pattern similarly well. All models are chemically sensible and contain chains of molecules connected by double hydrogen bonds. Models *A*1, *A*3 and *A*4 are quite similar and contain chains with steps, as in DCQ. Model *B*1 contains wavy chains. In model *C*1 the chains run in two different directions. Model *B*1 gives the best fit, but at the expense of double the number of atomic parameters. Model *B*1 was transformed from 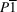 , *Z* = 2 to 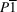 , *Z* = 1, yielding model *B*1*Z*1. After a careful Rietveld refinement the fit was quite good (Fig. 9 ▸). Nevertheless the hydrogen-bond topology remains questionable. Even with DFT-D calculations it remains unclear which is the correct structure. A detailed discussion, including Rietveld plots and figures of the structures, is provided in section S5 in the supporting information.

This example shows both the power and limitations of *FIDEL-GO*’s global optimization approach: even with very poor powder data, *FIDEL-GO* is able to find the crystal structures that match the diffraction data. However, it may happen that the Rietveld refinements do not allow the identification of the correct structure. In such cases, additional information is required, *e.g.* from electron diffraction (Gorelik *et al.*, 2009 ▸, 2021 ▸), elaborate solid-state NMR (Bryce & Taulelle, 2017 ▸; Schlesinger *et al.*, 2022 ▸) or PDF analyses (Billinge, 2019 ▸; Schlesinger *et al.*, 2021 ▸).

### 5.4. SDPD of CuCP by global optimization

Dichloro-bis(pyridine-*N*)copper(II) {[CuCl_2_(C_5_H_5_N)_2_]_*n*_, CuCP, Fig. 1 ▸} is a member of a series of coordination polymers which we reported recently (Krysiak *et al.*, 2014 ▸; Zhao *et al.*, 2017 ▸; Heine *et al.*, 2018 ▸, 2020 ▸). The compound consists of infinite copper–halogen chains of *trans*-edge-sharing distorted octahedra. It was used to test *FIDEL-GO*’s capabilities to work with different types of intramolecular degrees of freedom. The sample was of sufficient crystallinity, but the measured X-ray powder data suffered from a low signal-to-noise ratio. Therefore, the powder pattern was smoothed by *FIDEL-GO* using *PeakSearch* (Oishi-Tomiyasu, 2012 ▸).

**Figure 10:**
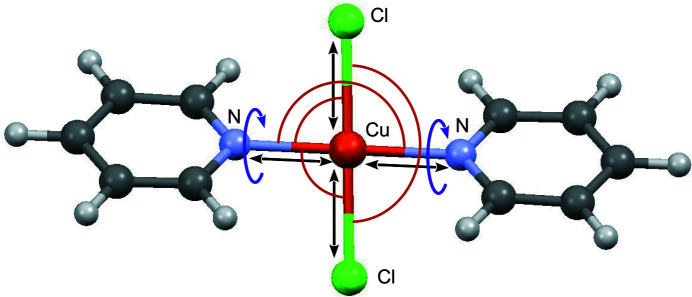
CuCP: a fragment on a general position with internal degrees of freedom for bond lengths (black), angles (red) and torsions (violet).

The molecular fragment CuCl_2_(C_5_H_5_N)_2_ is shown in Fig. 10 ▸. The geometry of the pyridine ligand and reasonable ranges for the Cu—N and Cu—Cl bond lengths were derived from similar complexes and *Mogul* statistics of structures in the CSD. Global optimization runs with the fragment CuCl_2_(C_5_H_5_N)_2_ on a general position and ten internal degrees of freedom (Fig. 10 ▸) were performed in space groups 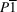 , *P*2_1_, *C*2/*c*, *P*2_1_/*c* and *P*2_1_2_1_2_1_. Additional runs with copper on a crystallographic inversion centre were performed in 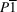 , *C*2/*c*, *P*2_1_/*c* and *Pbca* using the fragment CuCl(C_5_H_5_N) with four degrees of freedom, *i.e.* the Cu—N and Cu—Cl distances, the N—Cu—Cl angle and the rotation around the Cu—N bond (see Table S12 in the supporting information).

**Figure 11:**
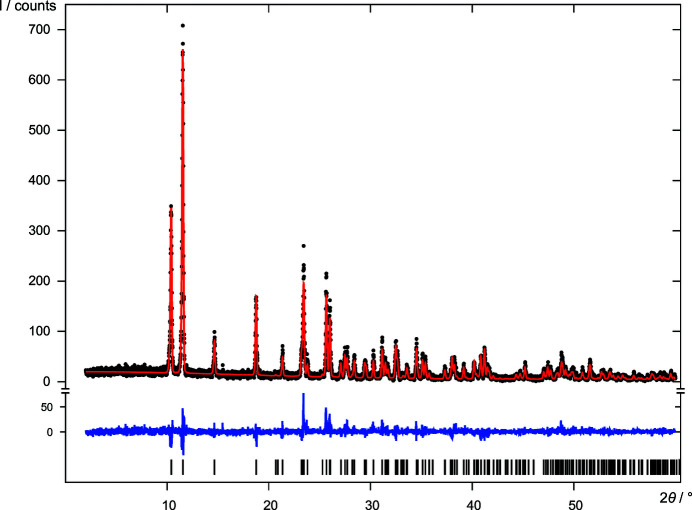
CuCP: final Rietveld refinement (UR) in *P*2_1_/*c*, *Z* = 2. For the diagram legend see Fig. 4 ▸.

Global optimization by *FIDEL-GO* resulted in a number of top ranking structure candidates in *P*2_1_/*c* (*Z*′ = 0.5) and *P*2_1_ (*Z*′ = 1). An overview of the best primary result structures (stage RE1) in each of the nine crystal symmetries is shown in Table S13 in the supporting information. The final results of the global optimization (stage RE2, Table 9 ▸) show the high reliability and accuracy of the *FIDEL-GO* fit. The three structures with the highest 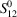 were subjected to automatic Rietveld refinements (stage AR, Table S14 in the supporting information), evaluated by DFT-D calculations (stage DO, Table S15 in the supporting information) and finally subjected to a user-controlled Rietveld refinement (stage UR) to the original unsmoothed powder data. All three structures proved to be practically identical. The evolution of the final structure is shown in Table 9 ▸ and the Rietveld plot in Fig. 11 ▸. Although the search fragments contained only an incomplete coordination sphere with only four of the six ligands, all final structures exhibited the correct polymeric structure with octahedrally coordinated Cu atoms.

The crystal structure determined here from scratch from powder data with a low signal-to-noise ratio is in excellent agreement with the structure determined by Morosin (1975 ▸) from Mo *K*α single-crystal data (see Table 9 ▸, and structure overlay in Fig. S5 in the supporting information). This demonstrates the capability and consistency of *FIDEL-GO*’s global optimization approach to structure determination from low-quality powder data, even with a flexible coordination complex.

## 6. Conclusions

A method for the *ab initio* determination of organic and metal–organic crystal structures from powder data without prior indexing has been developed and implemented in the program *FIDEL-GO*. The global optimization approach uses the similarity measure *S*_12_, which is based on weighted cross-correlation functions, for ranking, fitting and clustering of trial structures. SDPD from scratch requires only a reasonable molecular geometry and a general setup of the global search space in selected crystal symmetries. The unit-cell parameters, molecular position and orientation, and selected internal degrees of freedom are fitted simultaneously to the powder pattern.

In order to realize an efficient and effective exploration of the global search space, a hierarchical search strategy has been developed. The global optimization starts from a huge number of random trial structures and combines pre-selection by a rough comparison to the powder data with local optimizations of suitable candidates. The standard overall SDPD procedure of *FIDEL-GO* consists of three major steps: (i) the actual global optimization runs (GO), (ii) the re-evaluation of top ranking primary results by the best possible *FIDEL* fits (RE) followed by automatic Rietveld refinements (AR) of promising user-selected structures, and (iii) the final identification and refinement of one or several best matching structure candidates based on user-controlled Rietveld refinements (UR) and, optionally, DFT-D calculations (DO).

*FIDEL-GO*’s robust approach to pattern comparison and structure fitting is suitable for experimental patterns of very low quality and for nanocrystalline powders. With the implementation of the global optimization method and the integration of many auxiliary components, *FIDEL-GO* has evolved into an almost comprehensive application framework. The elaborate multi-step procedure can easily be adapted to a wide range of application scenarios in powder diffraction. By downscaling and specific configuration of the method, it is possible to make use of additional information and to adapt the method to the specific characteristics of a problem.

The method was successfully applied to the *ab initio* structure determination from unindexed powder data of (metal–)organic phases consisting of small to medium-sized rigid or moderately flexible molecules. It is already viable in terms of computing time on a standard PC. With the increasing performance of common equipment and the growing availability of distributed computing environments, SDPD from scratch by global optimization shall soon be a common option.

A remarkable aspect arising from the application of the method to SDPD from scratch using ‘problematic’ powder data is the challenge of the paradigm ‘one powder – one structure’. The global optimizations may yield several solutions with a similarly good fit to the experimental pattern. Even if the method does not provide a unique solution, the obtained structural models are very valuable. On the one hand they give a very good impression of the possible crystal structures. On the other hand there are many other analytical tools available to resolve which of the structures is the correct one, *e.g.* computational methods such as DFT-D (as shown here), specific modelling and refinement techniques (*e.g.* with respect to disordered structures) or complementary experimental approaches such as electron diffraction, solid-state NMR or vibrational spectroscopy.

In order to assess the method’s full scope and limitations, more rigorous evaluations on a broader scale have to be performed. This will include its application to a larger variety of actually existing examples, as well as systematic investigations based on specifically designed sets of simulated powder patterns. The method can complement existing approaches as a useful tool for the acquisition of structural information from powder diffraction data that is otherwise difficult or impossible to obtain and is in most cases discarded due to the lack of reliable indexing. Based on our experience with *FIDEL-GO* and the characteristics of the approach, we expect the method will also serve well for more flexible molecules, structures with *Z*′ > 1, disordered structures and phase-impure samples.

Finally, it should be noted that the similarity measure *S*_12_ can also be applied to pair distribution functions (Habermehl *et al.*, 2021 ▸). There, *S*_12_ and the structure solution approach of *FIDEL-GO* have been successfully used for the structure determination of organic compounds from scratch by a fit to the PDF without prior indexing (Schlesinger *et al.*, 2021 ▸).

## 7. Related literature

For further literature related to the supporting information, see Cheary & Coelho (1998 ▸), Huang *et al.* (2018 ▸) and Pawley (1981 ▸).

## Supplementary Material

Crystal structure: contains datablock(s) global, DFQ, DCQ. DOI: 10.1107/S2052520622001500/ra5106sup1.cif

Structure factors: contains datablock(s) DFQ. DOI: 10.1107/S2052520622001500/ra5106DFQsup2.hkl

Structure factors: contains datablock(s) DCQ. DOI: 10.1107/S2052520622001500/ra5106DCQsup3.hkl

Five models for DCDHQ in CIF format. DOI: 10.1107/S2052520622001500/ra5106sup4.txt

Additional figures and tables. DOI: 10.1107/S2052520622001500/ra5106sup5.pdf

CCDC references: 2150865, 2150866

## Figures and Tables

**Table 1:** Evolution of the SDPD of DFQ: number of structural models at successive levels of the overall procedure The numbers of random structures refer to trial structures with non-overlapping molecules within the given cell volume range. To test the procedure on different computer platforms, additional GO runs were performed for all crystal symmetries, in particular for the triclinic space groups. Runs with adapted settings were performed for *P*2_1_/*c* with *Z*′ = 0.5 and *Z*′ = 1 to verify that they yield the best results matching the powder data equally well. The numbers of Rietveld refined structures (AR, UR) are listed under the crystal symmetries from which they originated, irrespective of the space groups actually used in the refinement.

	Total	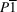	*P*1	*P*2_1_	*C*2/*m*	*P*2_1_/*c*	*P*2_1_/*c*	*C*2/*c*	*P*2_1_2_1_2_1_	*Pbca*
*Z*		1	1	2	2	2	4	4	4	4
*Z*′		0.5	1	1	0.25	0.5	1	0.5	1	0.5
Site symmetry		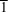	1	1	2/*m*	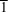	1	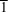	1	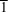
Fitted parameters		9	9	9	5	7	10	7	9	6
Global optimization runs (GO)	28	6	6	2	2	3	3	2	2	2
Random structures (GO2)	21 159 231	1 892 181	3 547 646	1 627 772	424 325	3 009 164	5 404 365	1 820 121	1 451 027	1 982 630
Local optimizations (GO3)	137 339	18 522	18 928	4707	6629	14 923	48 999	7393	8044	9194
Post-optimizations (GO4)	7828	1566	1800	450	650	875	737	650	450	650
Primary results (GO)	13 794	3810	4689	407	187	2247	906	974	214	360
Automatic evaluation of the primary results:										
Filtered/selection (RE1)	400	58	56	52	21	43	45	50	47	28
Re-evaluation fine fit (RE2)	122	42	40	2		8	30			
After evaluation and selection by the user:										
DFT-D geometry optimizations (DO)	5	1	1			1	2			
Rietveld refinements:										
Automatic (AR)	18	4	4			4	6			
User-controlled (UR)	6		1			2	3			

**Table 2:** DFQ: primary results of the global optimization by *FIDEL-GO* (stage RE1) and best structure of each crystal symmetry

Space group	*Z*	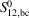	*V*/*Z* (Å^3^ mol^−1^)	*a* (Å)	*b* (Å)	*c* (Å)	α (°)	β (°)	γ (°)
*P*2_1_/*c*	4	0.9605	357.58	3.762	27.812	14.019	90	102.80	90
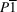	1	0.9481	359.14	3.675	7.072	14.166	98.90	90.80	98.78
*P*1	1	0.9481	359.14	3.675	7.072	14.166	98.90	90.80	98.78
*P*2_1_/*c*	2	0.9463	360.94	14.323	3.763	13.706	90	102.24	90
*P*2_1_	2	0.9293	355.86	7.014	27.981	3.714	90	102.43	90
*C*2/*c*	4	0.9259	376.72	14.490	3.842	28.890	78.03	78.55	75.84
*P*2_1_2_1_2_1_	4	0.9109	361.51	3.926	13.278	27.738	90	90	90
*Pbca*	4	0.8806	346.90	3.760	13.298	27.751	90	90	90
*C*2/*m*	2	0.8663	369.69	15.521	3.405	15.683	90	116.86	90

**Table 3:** SDPD of DFQ, structural models *A*–*D*: (*a*) final results of the global optimization, (*b*) automatic Rietveld refinements, models with *Z*′ = 0.5 refined in subgroups with *Z*′ = 1 or in *P*1, (*c*) DFT-D geometry optimizations, energies given relative to the lowest energy of all calculations, and (*d*) user-controlled Rietveld refinements with molecular geometry restraints derived from DFT-D For more details see Tables S2, S3, S4 and S5 in the supporting information. GoF stands for goodness of fit.

	Model	*A*	*B*	*C*	*D*
	Space group	*P*2_1_/*c*	*P*2_1_/*c*	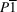	*P*2_1_/*c*
	*Z*	4	2	1	4
					
(*a*) *FIDEL-GO* global optimization (RE2)					
	Rank	1	2	8	34
	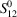	0.9891	0.9875	0.9840	0.9819
	*R*_wp_ (%)	18.69	20.02	22.55	29.88
	*V*/*Z* (Å^3^ mol^−1^)	358.87	358.92	369.82	356.38
					
(*b*) Automatic Rietveld refinement (AR) with *TOPAS*					
	Rank	6	1	8	10
	*R*_exp_ (%)	1.107	1.095	1.107	1.107
	*R*_wp_ (%)	10.888	7.484	11.966	12.822
	*R*_wp_′ (%)	20.659	14.021	22.419	23.162
	GoF	9.832	6.833	10.808	11.580
	*V*/*Z* (Å^3^ mol^−1^)	358.23	355.25	367.71	353.94
					
(*c*) DFT-D geometry optimization (DO) with *CASTEP*					
	Rank	1	2	4	3
Cell fixed	Δ*E* (kJ mol^−1^)	5.18	5.57	31.12	16.69
	*V*/*Z* (Å^3^ mol^−1^)	358.87	358.92	369.82	357.73
Cell optimized	Δ*E* (kJ mol^−1^)	0	0.54	24.56	13.89
	*V*/*Z* (Å^3^ mol^−1^)	336.7	337.2	346.4	344.8
					
(*d*) User-controlled Rietveld refinement (UR) with *TOPAS*					
	Rank	1	2	4	3
	*R*_exp_ (%)	1.276	1.280	1.280	1.276
	*R*_wp_ (%)	5.160	6.759	9.946	8.569
	*R*_wp_′ (%)	9.018	12.249	17.679	14.796
	GoF	4.045	5.279	7.770	6.718
	*V*/*Z* (Å^3^ mol^−1^)	358.54	358.33	361.44	359.62
	*a* (Å)	13.696	14.217	3.885	14.335
	*b* (Å)	3.768	3.768	7.033	3.773
	*c* (Å)	28.789	13.704	14.101	27.374
	α (°)	90	90	102.71	90
	β (°)	105.16	102.50	86.08	103.69
	γ (°)	90	90	105.94	90

**Table 4:** DCQ: final results of the global optimization by *FIDEL-GO* (stage RE2)

Rank	Space group	*Z*′	*W*_C_	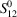	*V* (Å^3^ mol^−1^)	*a* (Å)	*b* (Å)	*c* (Å)	α (°)	β (°)	γ (°)
1	*P*1	1	43	0.9868	391.08	3.797	6.551	16.125	95.33	91.14	101.49
2	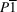	0.5	55	0.9868	391.38	3.797	6.553	16.131	95.31	91.15	101.46
3	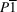	0.5	15	0.9809	350.56	3.829	5.921	16.121	96.32	91.12	104.94
4	*P*1	1	14	0.9807	350.94	3.821	5.935	16.112	96.12	91.36	104.72
5	*P*1	1	5	0.9803	388.68	3.715	6.735	16.195	98.32	93.49	103.10
											
10	*P*2_1_/*c*	1	4	0.9788	341.81	16.432	10.614	8.086	90	75.80	90
											
25	*P*2_1_2_1_2_1_	1	4	0.9770	345.00	4.078	10.584	31.971	90	90	90
											
60	*P*1	1	3	0.9703	378.40	3.664	6.478	16.177	95.36	93.75	96.91

**Table 5:** Stages in the evolution of the SDPD of DCQ for the structure that finally turned out to be the correct one The initial random trial structure (GO1–GO2) in *P*1 evolved through the fitting steps of the global optimization run (GO3–GO4) and went through automatic cell transformation and re-evaluation fine fit (RE). It was then transformed to 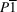 and subjected to the final user-controlled Rietveld refinement (UR). The similarity values *S*_12_(*l*) refer to the comparison of the simulated pattern to the background-corrected experimental pattern based on the 2θ comparison range set for the global optimization. 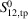 refers to the comparison of the powder pattern simulated by *TOPAS* and the experimental pattern. *E*_FF_ denotes the force-field energy.

	Random	Fast raw fit	Better fit 1	Better fit 2	Automatic transformation	Re-evaluation fit	Final Rietveld refinement	
	GO2	GO3	GO4	GO4	RE1	RE2		UR
Space group (*Z*)	*P*1 (*Z* = 1)	*P*1 (*Z* = 1)	*P*1 (*Z* = 1)	*P*1 (*Z* = 1)	*P*1 (*Z* = 1)	*P*1 (*Z* = 1)		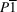 (*Z* = 1)
*l* (°)	1.0	0.72	0.58	0.43		0.1		
Similarity *S*_12_(*l*)	0.7331	0.8997	0.9615	0.9536		0.9714		
Reference similarity				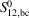	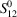	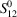		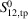
				0.9186	0.9836	0.9868		0.9969
*V*/*Z* (Å^3^ mol^−1^)	355.07	388.16	388.93	389.58	389.58	391.08		377.20
*a* (Å)	3.868	3.797	3.797	3.797	3.797	3.797		3.772
*b* (Å)	5.888	6.442	6.508	6.521	6.521	6.551		6.479
*c* (Å)	15.944	16.233	16.132	16.131	16.131	16.125		15.774
α (°)	85.99	86.53	85.30	85.04	94.96	95.33		93.74
β (°)	90.85	90.74	91.14	91.14	91.14	91.14		92.19
γ (°)	78.68	78.41	78.41	78.41	101.59	101.49		100.92
Δφ_*x*_ (°)	−15.9	−15.8	−24.3	−25.2			*R*_exp_ (%)	4.599
Δφ_*y*_ (°)	−66.5	−66.3	−64.7	−64.5			*R*_wp_ (%)	5.219
Δφ_*z*_ (°)	24.6	25.0	34.4	35.2			*R*_wp_′ (%)	8.438
*E*_FF_ (kJ mol^−1^)	−115.3		−207.7	−207.4			GoF	1.135

**Table 6:** DCQ: *FIDEL-GO* screening of CSP results by fitting to the experimental data, showing the best structure of each crystal symmetry

Space group	*Z*	Site symmetry	Screened	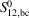	*V*/*Z* (Å^3^ mol^−1^)	*a* (Å)	*b* (Å)	*c* (Å)	α (°)	β (°)	γ (°)
*P*1	1	1	300	0.9193	389.91	3.801	6.514	16.135	94.92	91.16	101.39
*P*2/*c*	4	1	150	0.9173	468.30	7.348	3.976	64.427	90	95.60	90
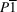	1	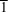	100	0.9161	391.19	3.818	6.515	16.115	94.92	90.63	101.48
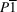	2	1	150	0.9003	385.30	6.825	7.458	16.520	100.02	95.98	109.11
*P*2_1_/*c*	2	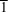	300	0.8889	366.41	16.488	3.538	12.895	90	103.05	90
*P*2_1_	2	1	300	0.8573	393.18	3.843	31.760	6.514	90	98.51	90
*P*2/*c*	2	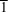	300	0.8460	374.82	16.241	6.360	7.327	90	97.88	90
*Pbca*	4	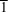	244	0.8176	274.20	5.049	6.729	32.284	90	90	90
*P*2_1_/*c*	4	1	100	0.7907	357.38	16.963	6.471	13.204	90	99.48	90
*P*2_1_2_1_2_1_	4	1	146	0.7208	384.58	7.086	12.869	16.871	90	90	90
*Pbca*	8	1	100	0.7089	385.53	6.854	6.841	65.782	90	90	90

**Table 7:** DCDHQ: final results of the global optimization by *FIDEL-GO* (stage RE2)

Rank	Model	Space group	*Z*	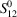	*R*_wp_ (%)	*V*/*Z* (Å^3^ mol^−1^)	*a* (Å)	*b* (Å)	*c* (Å)	α (°)	β (°)	γ (°)
1	*B*2	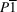	2	0.9879	17.070	418.88	4.118	12.991	16.668	70.22	88.68	86.95
2	*A*4	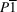	1	0.9875	18.114	375.90	3.702	6.586	15.696	86.82	88.54	79.68
3		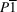	2	0.9873	18.074	405.89	6.487	7.973	15.934	80.70	86.58	88.39
4	*B*1	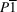	2	0.9872	18.031	413.22	4.062	13.016	16.696	109.33	92.95	95.18
5		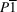	2	0.9872	17.077	391.81	6.121	8.175	16.208	103.66	94.70	92.61
6		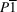	2	0.9872	20.831	391.79	3.851	13.003	16.704	109.27	93.78	94.36
7		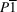	2	0.9871	17.055	404.26	7.272	7.759	15.679	89.25	88.75	66.08
8	*C*1	*P*2_1_/*c*	2	0.9870	15.872	418.65	6.494	4.097	31.531	90	93.52	90
9		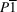	1	0.9868	17.956	417.35	4.103	6.486	15.799	86.91	83.81	88.96
10	*A*3	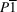	1	0.9867	17.981	421.88	4.152	6.577	15.698	86.69	88.45	80.38
11	*A*2	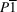	1	0.9867	18.127	382.63	3.770	6.601	15.677	92.86	90.44	100.88
12		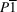	1	0.9867	19.585	380.40	3.741	6.641	15.710	86.72	88.74	77.51
13		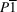	2	0.9866	18.870	405.08	6.973	7.715	15.823	93.02	95.13	106.50
14	*A*1	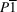	1	0.9865	17.955	380.17	3.747	6.572	15.683	92.79	90.29	99.71
15		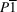	2	0.9864	18.645	373.15	6.923	6.988	16.000	83.32	80.55	78.87
16	*A*5	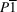	1	0.9863	18.300	410.34	4.037	6.483	15.788	92.84	96.02	91.02

**Table 8:** DCDHQ: user-controlled Rietveld refinements (stage UR) Models *B*1 and *A*3 started from the lowest-energy structures of the DFT-D calculations after re-adjustment of the unit-cell parameters by *FIDEL* fitted to the experimental pattern. Models *C*1 and *A*4 started from the final global optimization results (Table 7 ▸). Model *A*1 started from the best structural model of the automatic Rietveld refinement (see Table S10 in the supporting information) after (re)transformation from *P*1 to 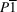 . Model *B*1*Z*1 started from *B*1 after transformation to 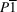 , *Z* = 1. The Rietveld refinement of *B*1*Z*1 was performed with different settings, hence the *R* and GoF values cannot be compared with the other refinements. Molecular geometry restraints were derived from DFT-D.

Model	Space group	*Z*	*R*_exp_ (%)	*R*_wp_ (%)	*R*_wp_′ (%)	GoF	*V*/*Z* (Å^3^ mol^−1^)	*a* (Å)	*b* (Å)	*c* (Å)	α (°)	β (°)	γ (°)
*B*1	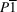	2	3.028	5.433	11.448	1.795	389.99	3.852	12.966	16.718	69.89	84.10	88.11
*C*1	*P*2_1_/*c*	2	3.152	9.489	21.314	3.010	421.70	6.508	4.113	31.564	90	93.27	90
*A*3	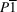	1	3.150	10.816	24.358	3.433	394.71	3.897	6.748	15.656	86.99	89.29	73.77
*A*4	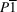	1	3.151	10.979	24.568	3.484	380.28	3.752	6.574	15.657	87.17	90.34	80.40
*A*1	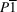	1	3.150	11.055	25.241	3.509	382.88	3.771	6.578	15.680	92.81	90.89	99.63
*B*1*Z*1	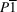	1	3.076	6.322	14.129	2.055	390.27	3.847	6.713	15.673	87.52	91.46	74.99

**Table 9:** CuCP: final results of the global optimization by *FIDEL-GO* (stage RE2) For the best structure candidate the automatic Rietveld refinement (AR) and the final Rietveld refinement (UR) after transformation to *P*2_1_/*c* (*Z* = 2) are shown, together with the published reference structure (CSD refcode PYRCUC02) after transformation from *P*2_1_/*n* (*Z* = 2) to *P*2_1_/*c* (*Z* = 2) for comparison. The 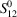 values given for the Rietveld refinements refer to the comparison of the powder pattern simulated by *TOPAS* to the smoothed experimental pattern (AR) or the original experimental data (UR).

Rank		Space group	*Z*′	*Z*	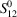	*V*/*Z* (Å^3^ mol^−1^)	*a* (Å)	*b* (Å)	*c* (Å)	β (°)
1		*P*2_1_	1	2	0.9758	282.24	3.861	8.592	17.026	91.98
	Automated Rietveld (AR)	*P*2_1_	1	2	0.9968	281.55	3.862	8.580	17.006	91.98
	Final Rietveld (UR)	*P*2_1_/*c*	0.5	2	0.9926	281.52	3.862	8.580	17.307	100.91
	Reference structure	*P*2_1_/*c*	0.5	2		279.25	3.848	8.560	17.268	100.89
2		*P*2_1_/*c*	0.5	2	0.9736	282.07	3.859	8.592	17.323	100.81
3		*P*2_1_/*c*	0.5	2	0.9730	282.19	3.859	8.598	17.319	100.84
4		*P*2_1_/*c*	1	4	0.9681	282.42	7.724	8.593	17.330	100.84
5		*P*2_1_	1	2	0.9617	277.74	8.597	17.029	3.795	91.11
6		*P*2_1_	1	2	0.9614	277.93	8.607	17.021	3.800	92.99
7		*P*2_1_/*c*	1	4	0.9611	278.04	7.592	17.040	8.598	90.99
