# Supplementary material for: Structure determination from unindexed powder data from scratch by a global optimization approach using pattern comparison based on cross-correlation functions
Source: Acta Crystallogr B Struct Sci Cryst Eng Mater. 2022 Mar 16;78(Pt 2):195–213. doi: 10.1107/S2052520622001500 (PMC9004021; doi:10.1107/S2052520622001500)
Supplement: Supplementary file 5 [file b-78-00195-sup5.pdf]

STRUCTURAL SCIENCE
CRYSTAL ENGINEERING
MATERIALS

Volume 78 (2022)

Supporting information for article:

Structure determination from unindexed powder data from scratch by a global optimization approach using pattern comparison based on cross-correlation functions

Stefan Habermehl, Carina Schlesinger and Martin U. Schmidt

Acta Crystallographica Section B
Structural Science, Crystal Engineering and Materials

Electronic Supplementary Information (ESI) - Habermehl *et al.* (2022): Structure determination from unindexed powder data from scratch by a global optimization approach using pattern comparison based on cross-correlation functions

S1. Powder diffraction details

In order to test and demonstrate the capabilities of the *FIDEL-GO* method, the application examples all started with problematic powder diffraction data. An overview of the XRPD measurements of the four samples is shown in Table S1. The samples of the quinacridone derivatives DFQ, DCQ and DCDHQ inherently suffer from small domain sizes. Only the diffraction measurement of DFQ can be regarded as a best-practice measurement for SDPD with standard laboratory equipment. The powder patterns of DCQ and DCDHQ are from routine measurements for phase identification. The test case for the known structure of CuCP was intentionally based on the example of a fast overview measurement.

Table S1
Details of the XRPD measurements in transmission mode with Cu $K\alpha_1$ radiation at room temperature on STOE STADI-P diffractometers with a curved Ge(111) monochromator and a linear position-sensitive detector.

Sample	2θ range / °	2θ stepsize / °	Max. intensity / counts
DFQ	2.00 - 79.99	0.01	94026
DCQ	3.00 - 33.98	0.02	3807
DCDHQ	3.00 - 33.98	0.02	6526
CuCP	2.00 - 59.99	0.01	708

S2. Rietveld refinements details

S2.1. Automatic Rietveld refinements (AR)

The automatic Rietveld refinements consist of a sequence of 7 *TOPAS* calls. The refinement sequence starts with two calls performing a Pawley fit (Pawley, 1981) to refine the background, described by 20 Chebyshev polynomial terms, the zero point error, the lattice parameters and a Gaussian and Lorentzian component convolution accounting for the crystallite size and strain broadening. The second run of the Pawley fit also includes the refinement of the peak width anisotropy, described by spherical harmonics of the 6th order. The subsequent Rietveld refinement of the structure is performed in 5 steps. In the first step only the scaling factor is refined. In the second step the background is refined additionally. In the third step one isotropic displacement parameter is refined for all non-hydrogen atoms, while still refining the scale factor and the background. Hydrogen atoms are included with an isotropic displacement parameter of 1.2 times the value for non-hydrogen atoms. The automatic refinements of CuCP included two additional isotropic displacement parameters for copper and chlorine. In the fourth step the refinement of the atomic coordinates is added, using a high penalty weighting for the molecular geometry restraints. In the last RV step the same variables are refined using a less stricter penalty weighting.

S2.2. User-controlled Rietveld refinements (UR)

The user-controlled refinements with *TOPAS* were always performed against the original (unsmoothed) powder data. They roughly followed the protocol described for the automatic refinement sequence or started from a certain stage of the automatic refinement sequence. At first, a Pawley refinement was carried out to refine the background, defined by 20 Chebyshev polynomial terms, the zero point error, the lattice parameters and the peak shape parameters, including peak asymmetry. The peak profile was described by the full axial divergence model using the method of Cheary & Coelho (1998). The parameters describing the background, the zero point and the peak shape were fixed at the beginning of the Rietveld refinement of the structural models. For DFQ and DCQ two isotropic displacement parameters for non-hydrogen atoms were refined, one for the halogen atom and one for carbon, nitrogen and oxygen atoms. The refinement of CuCP included two additional isotropic displacement parameters for copper and chlorine. It was always checked for preferred orientation. Preferred orientation was refined for DCQ, using the spherical harmonics description. In the investigation of the different structural models of DFQ (model A-D) and DCDHQ (models A1-C1) all models

for a given observed pattern were subjected to a strictly identical refinement procedure to ensure the comparability of the resulting structures. The final crystal structure (model B) of DFQ was further refined for the deposition in the CSD database. In the Rietveld refinement of CuCP the pyridine ring was restrained to be flat and C-C and C-H angles of the pyridine ring were restrained to 120°. At the end of the refinement of CuCP, the restraints for the Cu-Cl and Cu-N distances and all restraints on angles involving Cu atoms (Cl-Cu-N, Cu-N-C, etc.) were omitted, in order to search for possible Jahn-Teller-distortions of the octahedra.

S3. Structure determination of 4,11-difluoro-quinacridone (DFQ)

The following tables supplement the description of the SDPD of DFQ in section 5.1 of the paper. They provide a detailed overview of the structure candidates processed and evaluated in the stages from the global optimization to the final Rietveld refinement: the results of the global optimization after the automatic reevaluation (stage RE2, Table S2), the automatic Rietveld refinements (stage AR, Table S3), the DFT-D geometry optimizations (stage DO, Table S4) and the user-controlled Rietveld refinements (stage UR, Table S5).

Table S2
DFQ: final results of the global optimization with *FIDEL-GO* (stage RE2).

Rank	Model	Sp. gr.	Z'	R_{wp} / %	S_{12}^{0}	V/Z / Å^3/mol	a / Å	b / Å	c / Å	α / °	β / °	γ / °
1	A	$P2_1/c$	1	18.69	0.9891	358.87	13.685	3.763	28.794	90	104.50	90
2	B	$P2_1/c$	0.5	20.02	0.9875	358.92	14.299	3.763	13.691	90	102.94	90
3		$P2_1/c$	1	20.00	0.9870	354.23	13.544	3.758	28.406	90	101.47	90
4		$P2_1/c$	1	23.21	0.9863	352.61	13.516	3.754	28.280	90	100.60	90
5		$P2_1/c$	1	25.25	0.9860	352.70	13.513	3.754	28.288	90	100.55	90
6		$P2_1/c$	1	25.49	0.9856	359.37	13.717	3.765	28.745	90	104.46	90
7		$P2_1/c$	0.5	20.97	0.9851	358.40	14.314	3.763	13.662	90	103.05	90
8	C	$P\bar{1}$	0.5	22.55	0.9840	369.82	3.900	7.110	14.274	100.95	93.50	106.40
9		$P\bar{1}$	0.5	22.54	0.9839	369.53	3.896	7.111	14.264	100.79	93.47	106.47
10		$P\bar{1}$	0.5	25.96	0.9839	359.11	3.675	7.104	14.099	98.90	90.55	98.78
11		$P1$	1	22.78	0.9839	370.01	3.903	7.099	14.263	100.74	93.38	106.33
12		$P\bar{1}$	0.5	22.95	0.9838	370.32	3.904	7.101	14.264	100.72	93.29	106.34
13		$P\bar{1}$	0.5	23.18	0.9837	369.10	3.877	7.065	14.292	76.86	86.90	75.54
14		$P1$	1	23.05	0.9836	370.23	3.903	7.113	14.270	100.89	93.14	106.64
15		$P1$	1	22.43	0.9833	368.53	3.887	7.105	14.255	100.49	93.64	106.42
16		$P2_1/c$	1	25.43	0.9833	351.05	13.452	3.744	28.217	90	98.85	90
...												
28		$P\ 2_1/c$	0.5	19.85	0.9822	360.75	14.461	3.755	13.795	90	105.61	90
...												
34	D	$P2_1/c$	1	29.88	0.9819	356.38	14.245	3.759	27.263	90	102.43	90
...												
47		$P2_1/c$	0.5	20.87	0.9804	355.81	14.484	3.753	13.595	90	105.65	90
61		$P\bar{1}$	0.5	24.15	0.9796	371.22	3.990	6.702	14.131	81.81	82.99	88.17

Table S3
DFQ: automatic Rietveld refinements (stage AR) of selected structural models from the global optimization with *FIDEL-GO*. Models with Z'=0.5 have been refined in corresponding subgroups with Z'=1 or in $P1$.

Model	Sp. gr.	Z	R_{exp} / %	R_{wp} / %	R_{wp}' / %	GoF	V/Z / Å^3/mol	a / Å	b / Å	c / Å	α / °	β / °	γ / °
B	$P1$	2	1.095	7.484	14.021	6.833	355.25	14.391	3.763	13.603	90	105.33	90
	$P1$	2	1.095	8.149	14.740	7.440	356.53	14.199	3.766	13.655	90	102.44	90
	$P2_1/c$	4	1.107	10.055	19.091	9.080	353.25	13.561	3.763	28.301	90	101.96	90
	$P2_1/c$	4	1.107	10.516	19.092	9.497	352.35	13.521	3.761	28.232	90	100.95	90
	$P2_1/c$	4	1.107	10.699	19.459	9.662	352.25	13.520	3.760	28.248	90	101.14	90
A	$P2_1/c$	4	1.107	10.888	20.659	9.832	358.23	13.687	3.770	28.687	90	104.54	90
	$P2_1/c$	4	1.107	11.595	21.526	10.471	351.39	13.467	3.763	28.121	90	99.44	90
C	$P1$	1	1.107	11.966	22.419	10.808	367.71	3.896	7.043	14.201	102.26	86.80	105.06
	$P2_1/c$	2	1.113	12.102	22.476	10.871	359.16	14.402	3.762	13.770	90	105.68	90
D	$P2_1/c$	4	1.107	12.822	23.162	11.580	353.94	14.148	3.764	27.144	90	101.66	90
	$P1$	1	1.107	12.407	23.520	11.207	358.19	3.674	7.099	14.062	98.83	91.38	98.318
	$P1$	1	1.107	12.686	24.357	11.458	368.30	3.904	7.090	14.194	79.78	86.73	106.50
	$P1$	1	1.107	12.995	24.722	11.738	368.74	3.905	7.097	14.209	100.57	93.65	106.33
	$P2_1/c$	2	1.113	13.617	27.137	12.232	356.32	14.220	3.771	13.625	90	102.72	90
	$P1$	1	1.107	14.263	28.678	12.883	368.42	3.897	7.096	14.216	79.47	86.13	106.12
	$P\bar{1}$	1	1.113	16.846	29.905	15.135	372.27	4.013	6.696	14.074	82.23	96.42	92.12
	$P1$	1	1.107	14.729	30.284	13.304	368.51	3.902	7.084	14.204	102.63	86.27	105.88
	$P1$	1	1.107	17.055	34.873	15.405	366.21	3.885	7.110	14.405	103.70	93.78	106.79

Table S4
DFQ: DFT-D geometry optimizations of selected structural models. *CASTEP* calculations were performed with fixed cell dimensions at first and including the optimization of the lattice parameters in the second step. Energies given are relative to the lowest energy of all calculations.

Structure candidate			Cell optimized		Cell fixed							
Model	Sp. gr.	Z'	ΔE / kJ/mol	V/Z / Å^3/mol	ΔE / kJ/mol	V / Å^3/mol	a / Å	b / Å	c / Å	α / °	β / °	γ / °
A	$P2_1/c$	1	0	336.7	5.18	358.87	13.685	3.763	28.794	90	104.50	90
B	$P2_1/c$	0.5	0.54	337.2	5.57	358.92	14.299	3.763	13.691	90	102.94	90
C	$P\bar{1}$	0.5	24.56	346.4	31.12	369.82	3.898	7.110	14.274	100.95	93.50	106.40
D	$P2_1/c$	1	13.89	344.8	16.69	357.73	14.335	3.759	27.264	90	103.08	90
	$P1$	1	25.12	344.5	31.60	369.53	3.896	7.111	14.264	100.79	86.53	106.47

Table S5
DFQ: user-controlled Rietveld refinements of selected structural models (stage UR) under strictly identical conditions and final refinement of the submitted structure (model B). Molecular geometry restraints were derived from DFT-D calculation.

Model	Sp. gr.	Z'	R_{exp} / %	R_{wp} / %	R_{wp}' / %	GoF	V/Z / Å^3/mol	a / Å	b / Å	c / Å	α / °	β / °	γ / °
A	$P2_1/c$	1	1.276	5.160	9.018	4.045	358.54	13.696	3.768	28.789	90	105.16	90
	$P\ 2_1/c$	1	1.276	6.442	11.581	5.050	354.47	13.589	3.763	28.346	90	101.97	90
B	$P2_1/c$	0.5	1.280	6.759	12.249	5.279	358.33	14.217	3.768	13.704	90	102.50	90
	$P\ 2_1/c$	0.5	1.280	7.486	14.075	5.847	357.46	14.201	3.767	13.682	90	102.37	90
D	$P2_1/c$	1	1.276	8.569	14.796	6.718	359.62	14.335	3.773	27.374	90	103.69	90
C	$P\bar{1}$	0.5	1.280	9.946	17.679	7.770	361.44	3.885	7.033	14.101	102.71	86.08	105.94
B final	$P2_1/c$	0.5	1.466	6.452	14.425	4.402	359.08	14.217	3.768	13.721	90	102.30	90

S4. Structure determination of 2,9-dichloro-quinacridone (DCQ)

The SDPD of DCQ by global optimization (section 5.2.1 of the paper) is complemented by an overview of the top ranking primary results of the global optimizations in all investigated crystal symmetries (stage RE1, Table S6). The crystal structure of the best candidate from the global optimization is compared to the corresponding crystal structure after the DFT-D calculation in Fig. S1.

For the SDPD of DCQ by screening of CSP results (section 5.2.1 of the paper) an overview of the crystal structure prediction results is provided in Table S7.

Table S6
DCQ: primary results of the global optimization with *FIDEL-GO* (stage RE1). Best structure of each crystal symmetry.

Sp. gr.	Z	$S^0_{12,\mathrm{bc}}$	V/Z / Å^3/mol	a / Å	b / Å	c / Å	α / °	β / °	γ / °
$P1$	1	0.9186	389.58	3.797	6.521	16.131	94.96	91.14	101.59
$P2_12_12_1$	4	0.9176	389.94	3.721	6.530	64.184	90	90	90
$P\bar{1}$	1	0.9165	388.45	3.790	6.507	16.135	94.70	91.52	101.32
$P2_1/c$	4	0.8977	384.74	16.051	7.612	12.628	90	94.10	90
$P2_1$	2	0.8944	341.12	4.228	10.148	15.926	90	93.32	90
$P2_1/c$	2	0.8862	404.96	3.653	6.951	32.250	90	98.48	90
$C2/c$	4	0.8747	337.61	11.698	3.614	32.219	90	97.57	90
$C2/m$	2	0.8581	353.83	6.891	6.430	16.061	90	96.07	90
$Pbca$	4	0.8272	352.02	6.426	6.880	31.847	90	90	90

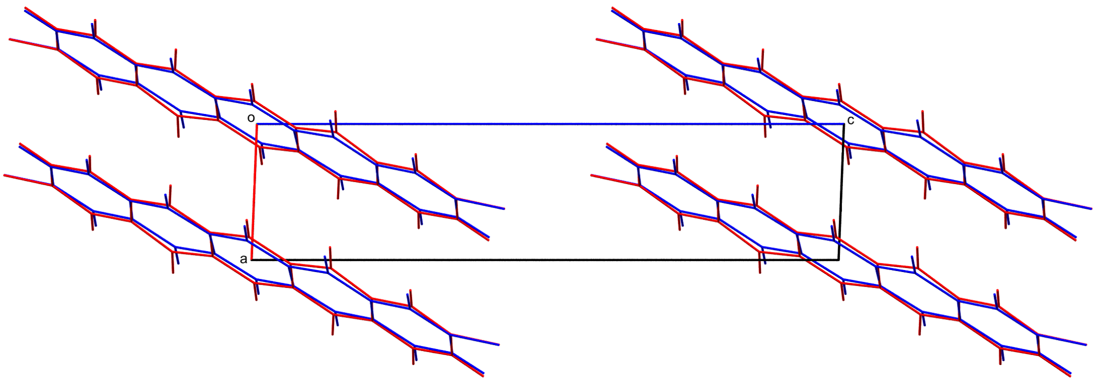

Figure S1
DCQ: structure overlay of the best candidate from the global optimization before (blue) and after (red) the DFT-D geometry optimization with fixed cell, view along [010].

Table S7
DCQ: crystal structure prediction by lattice energy minimization with *CRYSCA*. Lowest energy structure in each crystal symmetry, ranked by energy relative to the lowest energy of all calculations.

Sp. gr.	Z	Z'	Site symm.	CSP results	ΔE / kJ/mol	V/Z / Å^3/mol	a / Å	b / Å	c / Å	α / °	β / °	γ / °
$P1$	1	1	1	500	0	408.35	4.156	6.879	14.640	82.06	86.37	80.35
$P\bar{1}$	1	0.5	$\bar{1}$	118	4.44	410.59	3.778	7.033	15.617	91.24	91.12	98.14
$P\bar{1}$	2	1	1	157	6.88	420.43	7.428	8.483	14.684	90.87	100.28	111.97
$P2_1/c$	2	0.5	$\bar{1}$	585	7.81	405.90	7.337	17.347	7.196	90	117.58	90
$P2_1$	2	1	1	318	8.85	419.03	7.421	6.700	16.856	90	89.60	90
$Pbca$	4	0.5	$\bar{1}$	244	11.11	414.78	7.466	6.497	34.202	90	90	90
$Pbca$	8	1	1	112	11.38	408.64	7.454	6.695	65.511	90	90	90
$P2_1/c$	4	1	1	128	12.61	412.17	33.370	7.403	6.765	90	99.43	90
$P2/c$	2	0.5	$\bar{1}$	1190	13.05	415.20	6.361	3.899	37.649	90	117.21	90
$P2_12_12_1$	4	1	1	146	16.69	428.26	7.457	31.898	7.202	90	90	90
$P2/c$	4	1	1	179	21.20	459.22	43.822	7.513	6.917	90	126.23	90

S5. Structure determination of 2,9-dichloro-6,13-dihydro-quinacridone (DCDHQ)

The crystal symmetries under investigation in the global optimization runs for DCDHQ (section 5.3 of the paper) are documented in Table S8. An overview of the top ranking primary results of the global optimizations in all crystal symmetries (stage RE1) is shown in Table S9. Table S10 lists the results of the automatic Rietveld refinements (stage AR) and the results of the DFT-D geometry optimizations (stage DO) are documented in Table S11.

The crystal structures and the corresponding Rietveld plots of the structural models B1Z1, B1, A3 and C1 are shown in Fig. S2. A detailed discussion of the structure candidates is provided in §S5.2.

Table S8
DCDHQ: selection of the crystal symmetries for the global optimization. For setups of planar molecules (point group C_{2h}) with Z'=0.5 the inversion center of the molecule has been positioned at the crystallographic inversion center using a dummy atom.

Crystal system	Space group	Z	Z'	Site symm.	Intramol. DOFs	Fitted parameters
Triclinic	$P\bar{1}$	1	0.5	$\bar{1}$	0	9
		2	1	1	2	14
Monoclinic	$P2_1$	2	1	1	2	11
	$C2/c$	8	1	1	2	12
	$P2_1/c$	2	0.5	$\bar{1}$	0	7
		4	1	1	2	12
Orthorhombic	$P2_12_12_1$	4	1	1	2	11
	$Pbca$	8	1	1	2	11
	$Pna2_1$	4	1	1	2	11

Table S9
DCDHQ: primary results of the global optimization with*FIDEL-GO* (stage RE1). Best structure of each crystal symmetry.

Sp. gr.	Z	opt. runs	$S^0_{12,\mathrm{bc}}$	V/Z / Å^3/mol	a / Å	b / Å	c / Å	α / °	β / °	γ / °
$P\bar{1}$	2	4	0.9547	417.69	4.118	12.973	16.657	70.09	88.68	86.93
$P2_1/c$	2	4	0.9528	417.56	6.487	4.097	31.475	90	93.35	90
$P\bar{1}$	1	4	0.9521	378.80	3.743	6.601	15.656	86.95	89.41	78.74
$C2/c$	8	4	0.9475	285.99	12.943	5.645	31.346	90	92.54	90
$P2_1/c$	1	4	0.9422	390.68	6.622	62.731	3.769	90	93.58	90
$P2_1$	2	2	0.9330	459.39	4.135	31.274	7.189	90	98.74	90
$Pbca$	8	1	0.9284	379.76	14.205	31.327	6.827	90	90	90
$P2_12_12_1$	4	2	0.9261	419.09	7.104	7.527	31.350	90	90	90
$Pna2_1$	4	2	0.9234	421.34	62.767	3.777	7.110	90	90	90

Table S10
DCDHQ: automatic Rietveld refinements (stage AR) of selected structural models from the global optimization by *FIDEL-GO*, ranked by R_{wp}'. Models with Z'=0.5 have been refined in corresponding subgroups with Z'=1.

Model	Sp. gr.	Z	R_{exp} / %	R_{wp} / %	R_{wp}' / %	GoF	V/Z / Å^3/mol	a / Å	b / Å	c / Å	α / °	β / °	γ / °
A1	$P1$	1	2.988	7.781	20.386	2.604	382.12	3.775	6.577	15.643	87.33	89.02	99.87
A2	$P1$	1	2.988	7.846	20.388	2.626	382.38	3.778	6.583	15.642	92.66	89.05	79.80
B1	$P\bar{1}$	2	2.988	8.637	21.282	2.891	414.44	16.634	4.095	13.976	111.94	107.54	92.42
	$P1$	1	2.988	7.919	21.318	2.650	394.48	3.899	6.729	15.638	87.13	89.62	74.32
C1	$P2_1$	2	2.990	8.268	21.430	2.765	417.07	6.480	4.118	34.471	90	114.93	90
A3	$P1$	1	2.988	7.869	21.659	2.633	416.23	4.140	6.553	15.571	92.89	89.04	99.32
A4	$P1$	1	2.988	8.287	21.930	2.773	375.06	3.710	6.587	16.153	84.68	75.34	79.56
	$P1$	1	2.988	8.655	21.964	2.897	378.09	3.736	6.653	15.650	93.24	88.10	103.13
	$P1$	1	2.988	8.314	22.144	2.782	428.40	4.236	6.493	15.689	86.83	94.83	93.92
	$P1$	1	2.988	8.415	22.679	2.816	426.14	4.205	6.501	15.667	86.91	87.56	85.67
B2	$P\bar{1}$	2	2.988	8.875	23.338	2.970	413.34	4.082	13.422	16.637	71.26	91.95	74.96
A5	$P1$	1	2.988	9.060	24.889	3.032	410.59	7.689	4.054	15.738	96.09	89.13	122.57
	$P1$	1	2.988	9.006	25.071	3.014	416.16	4.111	6.479	15.741	92.95	83.69	90.97
	$P1$	1	2.988	9.278	25.701	3.105	436.99	4.320	6.485	15.716	87.49	95.73	87.08
	$P1$	1	2.988	9.475	26.155	3.171	418.02	4.126	6.480	15.737	87.04	95.80	90.93
	$P\bar{1}$	2	2.988	11.785	29.670	3.944	368.30	6.958	7.006	17.195	72.02	67.97	79.24

Table S11
DCDHQ: DFT-D geometry optimizations of selected structural models (stage DO). *CASTEP* calculations were performed with fixed cell dimensions (a) at first and including the optimization of the lattice parameters (b) in the second step. Models are ranked by molar energies for each step. Energy values given are relative to the lowest energy of all calculations.

Model	Sp. gr.	Z	ΔE / kJ/mol	V/Z / Å^3/mol	a / Å	b / Å	c / Å	α / °	β / °	γ / °
a) Cell fixed:										
B1	$P\bar{1}$	2	21.71	414.45	4.062	13.020	16.729	109.30	92.95	94.92
A2	$P\bar{1}$	1	27.96	381.80	3.770	6.601	15.633	92.73	90.36	100.73
A1	$P\bar{1}$	1	28.62	378.89	3.747	6.572	15.635	92.79	90.29	99.81
A3	$P\bar{1}$	1	33.47	420.87	4.152	6.577	15.668	86.56	88.45	80.25
A5	$P\bar{1}$	1	35.13	410.27	4.037	6.483	15.800	93.04	96.42	91.02
B2	$P\bar{1}$	2	36.03	419.14	4.118	13.418	16.670	71.37	91.32	75.20
C1	$P2_1/c$	2	40.47	417.56	6.487	4.097	31.475	90	93.35	90
A4	$P\bar{1}$	1	142.73	374.56	3.702	6.586	16.179	84.57	75.22	79.49
b) Cell optimized:										
A3	$P\bar{1}$	1	0	367.92	3.697	6.596	15.563	92.11	88.94	104.00
B1	$P\bar{1}$	2	1.07	366.61	3.683	12.777	16.680	110.15	93.98	92.40
A5	$P\bar{1}$	1	1.60	368.79	3.690	6.620	15.594	87.85	88.23	75.70
A2	$P\bar{1}$	1	10.21	367.29	3.626	6.626	15.727	87.36	88.729	103.21
A1	$P\bar{1}$	1	10.38	367.31	3.625	6.616	15.718	92.53	88.592	77.40
A4	$P\bar{1}$	1	12.68	374.30	3.639	6.697	17.153	100.95	70.42	107.24
C1	$P2_1/c$	2	17.19	378.19	6.779	3.650	30.572	90	90.40	90
B2	$P\bar{1}$	2	19.41	383.76	4.5708	13.240	14.585	67.07	89.65	72.19

Model B1Z1

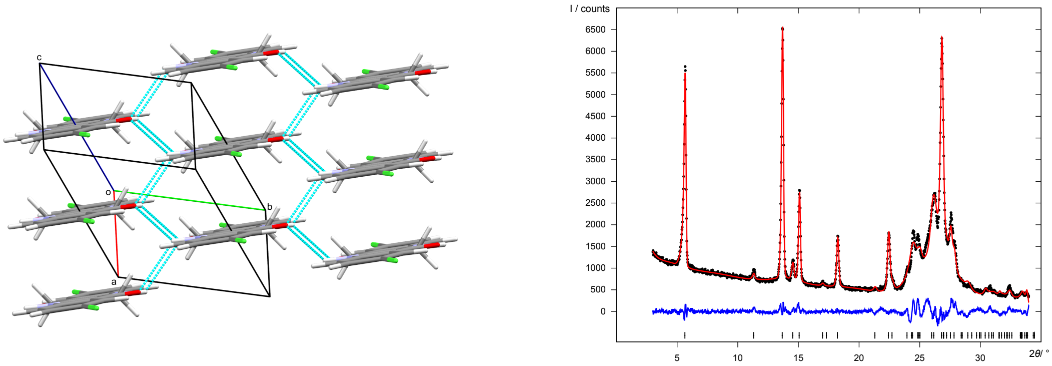

Model B1

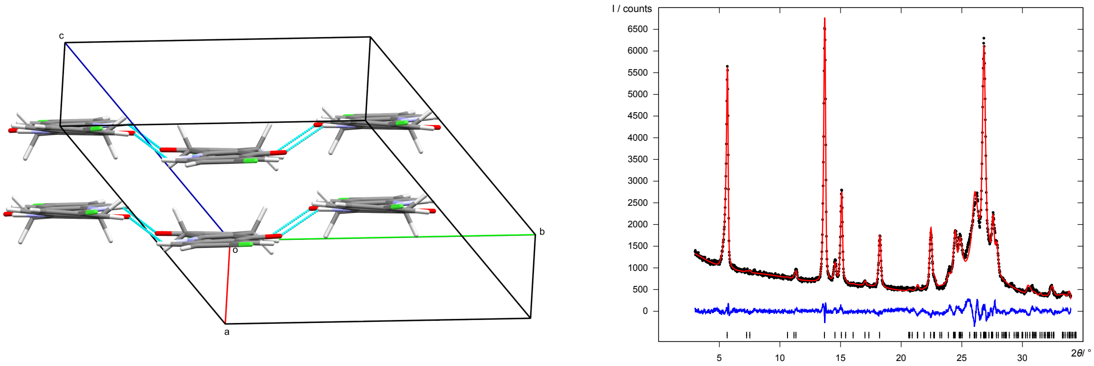

Model A3

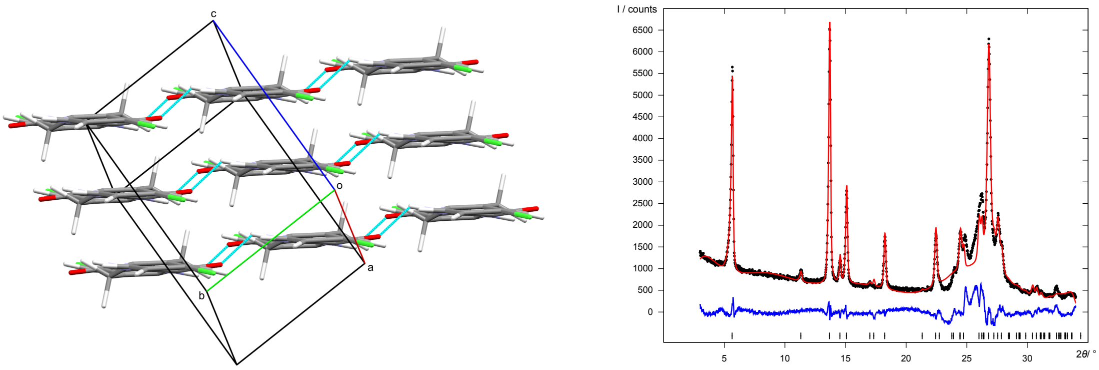

Model C1

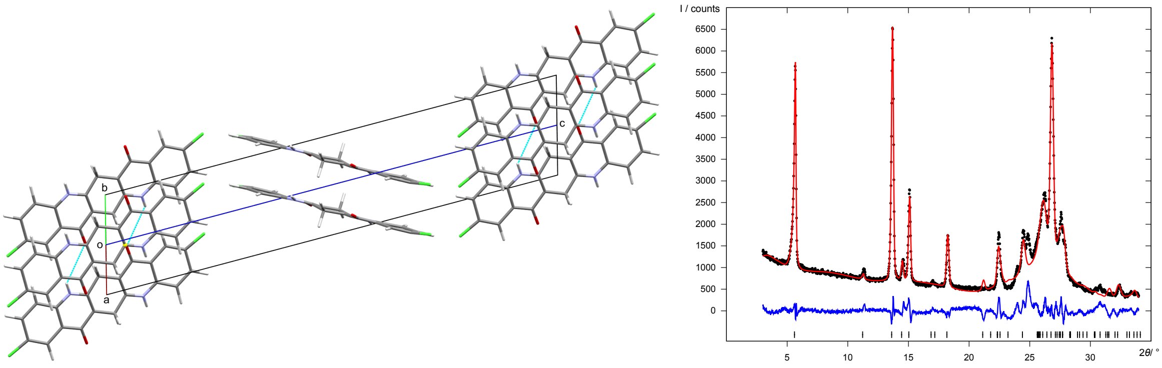

Figure S2
DCDHQ: crystal structures and Rietveld plots of the structural models B1Z1, B1, A3 and C1.

S5.1. On the molecular conformation of DCDHQ

Before the *FIDEL* calculations, we investigated the possible molecular conformations of DCDHQ by searches in the Cambridge Structural Database (CSD). The first search with the fragment F1 (Fig. S3) did not yield any crystal structure containing this fragment. The compounds F2 (Fig. S3) with methyl, ethyl, butyl or octyl groups are all planar (CSD refcodes TUCBOL, TUCBUR, TUCCAY, TUICCEC, PILLAY) (Huang *et al.*, 2018).

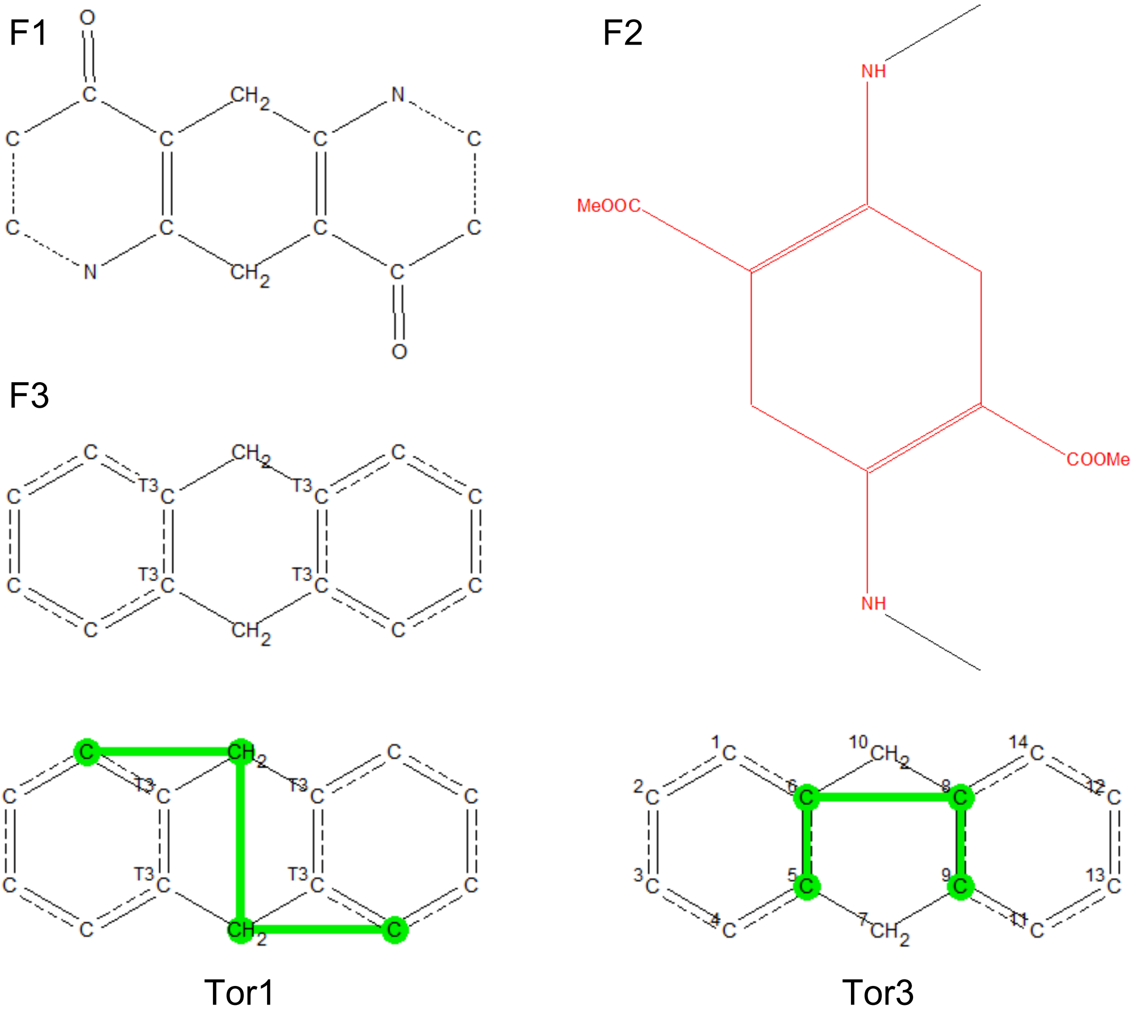

Figure S3
DCDHQ conformation, CSD searches: Definitions of search fragments F1, F2 and F3, and torsion angles Tor1 and Tor3. T3 denotes an atom with three bonds.

The next search was carried out with the fragment F3 (Fig. S3). Two torsion angles Tor1 and Tor3 (Fig. S3) were defined, that correspond to a tilting and a twisting of the molecule. The majority of these compounds is not planar. The distribution of the torsion angle Tor1 is shown in Fig. S4a.

9,10-Dihydro-anthracene itself (CSD refcode DITBOX01) has in the solid state a dihedral angle between the two benzene planes of 36.5° (corresponding to a torsion angle Tor1 of 143.5°). In contrast, the distribution of Tor3 is quite narrow (Fig. S4b).

These data show, that DCDHQ may be planar or tilted. In the *FIDEL* calculations, we allowed the molecule to tilt and to twist.

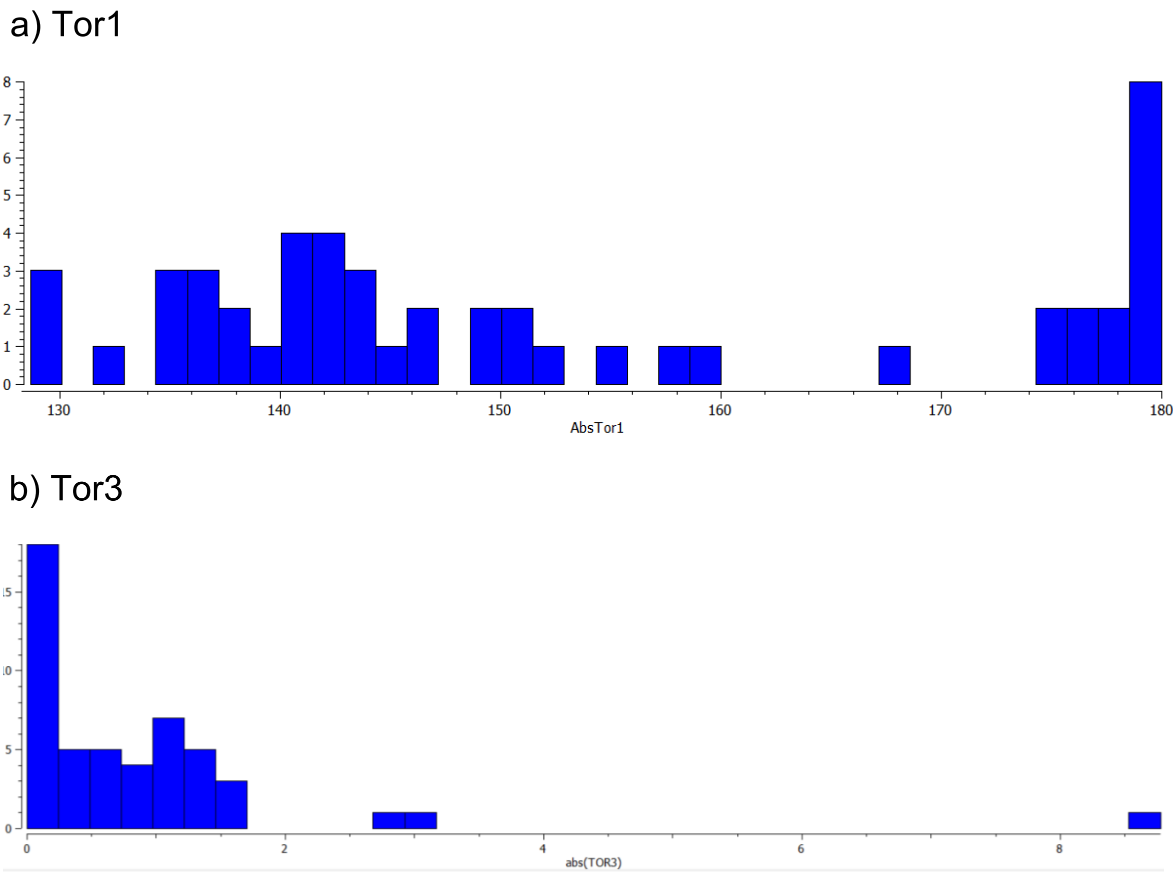

Figure S4
DCDHQ conformation, CSD search for fragment F3: Distribution of torsion angles (a) Tor1 and (b) Tor3.

S5.2. Detailed discussion on DCDHQ

The results of the user-controlled Rietveld refinements of the models A1, A3, A4, B1 and C1 are listed in Table 8 in the paper. The structure refinements all suffer to some degree from considerable drawbacks in terms of the difference curve, unusual deviations in the molecular geometry or relatively high errors of the refined parameters. The refinement of the model B1 (Z=2) led to a best possible result of the attempt to determine the structure from the "real life" powder pattern under investigation. This is supported by the best of all difference curves achieved, the low energies of the geometry optimizations of B1, a quite typical molar volume and the plausibility of the packing motive of the molecules (Fig. S2).

However, there is by no means sufficient evidence to prove the existence of a wavy chain structure in $P\bar{1}$ with Z=2. On the contrary, the most likely assumption on the crystal structure of DCDHQ that can be drawn from this investigation is, that the dihydro-compound crystallizes in $P\bar{1}$ (Z'=0.5), just like other triclinic structures of un- or 2,9-disubstituted quinacridones, in particular the α- and γ-phase of DCQ (see section 5.2 in the paper). This assumption is heavily supported by the DFT-D calculations (Table S11), that clearly show, that there is no significant advantage of the crystal symmetry with Z'=1 and that a packing in $P\bar{1}$ (Z'=0.5) is practically equivalent in terms of lattice energy and molar volume. In the resulting structure of the refinement of B1 there is a tilt of about 7° between two fairly planar halves of the molecule. Cell transformation of the structure with b'=a/2+b/2 led to a structural model with two of these tilted molecules to both sides of a plane that would represent the position of a fully planar molecule in $P\bar{1}$ (Z'=0.5), with the terminal chorine atoms at a mean distance of 0.14 Å and the sp^3-carbons in the central ring at a mean distance of 0.48 Å from the plane. While none of the quantum mechanical calculations gives rise to the expectation of a significant deviation from full planarity of the molecule, there may exist a non-neglectable tendency towards a slight tilt or twist of the molecules in the solid phase, leading to deviations from the perfect symmetry $P\bar{1}$ (Z'=0.5), e.g. a structure exhibiting orientational disorder. Two structure candidates selected for the user-controlled Rietveld refinement started from the primary results of the top ranking candidates of the global optimization by *FIDEL-GO* in $P2_1/c$ (Z'=0.5, model C1) and $P\bar{1}$ (Z'=0.5, model A4) (see Table 7 in the paper).

The packing index (PI) of the structural models, calculated by *PLATON* (Spek, 2009), has also been evaluated and compared to similar compounds. The PI of the structural models B1 and A3 converged to a value of about 80% on full DFT-D geometry optimization, while the PI of their Rietveld refinement results is at about 75%. This is in perfect agreement with known structures of

unsubstituted and disubstituted quinacridones, where the corresponding PI values span from 72.4% for unsubstituted γ-quinacridone up to 82.4% for a fully geometry optimized structure of 2,9-dimethyl-quinacridone. The structure candidate C1 in $P2_1/c$ (Z'=0.5) can be regarded as very unlikely, considering that all of its variants under investigation exhibited PI values of about 70-71%, more than 1% below the PI of any known crystal structure found so far for the compound class. The highest PI values of structural models, that were at least roughly considerable in the light of the DFT-D calculations and the Rietveld refinements for the room temperature structure, were found for three models in $P\bar{1}$ (Z'=0.5, models A1, A2 and A4). Their PI in the range 76.8-77.4% indicates a low likelihood of being a reasonable and correct representation of the crystal structure of DCDHQ, also taking into account the PI value 76.6% of the α-phase of DCQ (section 5.2 in the paper).

The powder pattern of DCDHQ came from a routine measurement of an intermediate in the industrial synthesis of DCQ. The sample had not been subjected to recrystallization or other attempts to increase its purity and crystallinity before the measurement. Accordingly, the experimental data probably suffer at least to some degree from various conditions, that inevitably limit the possibility of a fully satisfactory structure determination, such as chemical impurities, the minor presence of other phases and disturbances of order in the crystalline domains of the sample. Although the attempted SDPD was not able to yield a fully satisfactory and decisive result, it shows very well both the capabilities and the limitations of the global optimization approach of *FIDEL-GO* and its general framework for SDPD.

Finally, the structure B1 ($P\bar{1}$, Z=2) was transformed to $P\bar{1}$, Z=1 by $a'=a$, $b'=a/2+b/2$, $c'=a/2+b/2+c$ (model B1Z1). The subsequent user-controlled Rietveld refinement of B1Z1 was carried out in a different way than that of A1, A3, A4, B1 and C1 (15 instead of 10 background parameters; peak-width anisotropy described by spherical harmonics of the 2nd instead of the 4th order; B_{iso}(C, N, O) fixed to 3.0 and B_{iso}(Cl, H) fixed to 3.6; etc.). The resulting structure (Fig. S2) differs from structure A3 by a rotation of the molecules around their long axis, leading to a different hydrogen bond system: whereas A3 contains chains with steps, the molecules in B1Z1 exhibit bifurcated hydrogen bonds to four neighbouring molecules. All N···O distances are equal (3.32 Å). Such a packing has not been observed for any other quinacridone derivative. Due to the limited information content of the powder data, it remains unclear, if the structure actually exhibits these bifurcated hydrogen bonds or consists of chains with steps, as found in several other quinacridone derivatives, or even of wavy chains, as shown in Fig. S2.

S6. Structure determination of dichloro-bis(pyridine-N)-copper(II) (CuCP)

The crystal symmetries under investigation in the global optimization of CuCP (section 5.4 of the paper) are documented in Table S12. An overview of the top ranking primary results of the global optimizations in all crystal symmetries (stage RE1) is shown in Table S13. The automatic Rietveld refinements (stage AR) are documented in Table S14 and the DFT-D geometry optimizations (stage DO) in Table S15. The excellent agreement of the published single crystal structure and the crystal structure from the SDPD with *FIDEL-GO* is illustrated by the structure overlay in Fig. S5.

Table S12
CuCP: selection of the crystal symmetries for the global optimization. For setups with Z'=0.5 the metal atom has been fixed at the crystallographic inversion center.

Crystal system	Space group	Z	Z'	Site symm.	Internal DOFs	Fitted parameters
Triclinic	$P\bar{1}$	1	0.5	$\bar{1}$	4	13
		2	1	1	10	19
Monoclinic	$P2_1$	2	1	1	10	19
	$C2/c$	4	0.5	$\bar{1}$	4	11
		8	1	1	10	20
	$P2_1/c$	2	0.5	$\bar{1}$	4	11
		4	1	1	10	20
Orthorhombic	$P2_12_12_1$	4	1	1	10	19
	$Pbca$	4	0.5	$\bar{1}$	4	10

Table S13
CuCP: primary results of the global optimization (stage RE1). Best structure of each crystal symmetry.

Sp. gr.	Z	$S^0_{12,\mathrm{bc}}$	V/Z / Å^3/mol	a / Å	b / Å	c / Å	α / °	β / °	γ / °
$P2_1/c$	2	0.9728	281.28	3.855	8.584	17.308	90	100.84	90
$P2_1/c$	4	0.9662	282.29	7.724	8.589	17.338	90	100.99	90
$P2_1$	2	0.9648	282.03	3.861	8.585	17.024	90	91.84	90
$C2/c$	8	0.9536	305.51	11.742	12.421	16.766	90	91.78	90
$P\bar{1}$	1	0.9482	277.35	3.799	8.579	9.519	116.48	90.78	92.09
$C2/c$	4	0.9414	313.82	9.485	17.016	8.593	90	115.16	90
$P\bar{1}$	2	0.9394	309.79	8.324	8.974	9.770	112.81	102.04	102.85
$P2_12_12_1$	4	0.9232	307.76	8.418	8.604	16.998	90	90	90
$Pbca$	4	0.8976	290.44	16.935	3.992	17.186	90	90	90

Table S14
CuCP: automatic Rietveld refinements (stage AR) in $P2_1$, Z=2 vs. smoothed powder pattern.

Model	R_{exp} / %	R_{wp} / %	R_{wp}' / %	GoF	V/Z / Å^3/mol	a / Å	b / Å	c / Å	β / °
A1	2.372	7.935	8.866	3.346	281.55	3.862	8.580	17.308	100.91
B1	2.372	8.939	10.012	3.769	281.58	3.862	8.579	17.309	100.90
B2	2.372	9.765	10.988	4.118	281.57	3.862	8.579	17.309	100.90

Table S15
CuCP: DFT-D geometry optimizations (stage DO) of selected structural models in $P2_1/c$, Z=2. *CASTEP* calculations were performed with fixed cell dimensions at first and including the optimization of the lattice parameters in the second step. Energies shown are relative to the lowest energy of all calculations.

Model	Cell optimized		Cell fixed					
	ΔE / kJ/mol	V/Z / Å^3/mol	ΔE / kJ/mol	V/Z / Å^3/mol	a / Å	b / Å	c / Å	β / °
A1	0	540.68	2.43	563.11	3.862	8.580	17.308	100.91
B1	0.24	539.02	3.64	564.72	3.864	8.592	17.320	100.86
B2	not done	not done	2.47	563.15	3.862	8.579	17.309	100.90

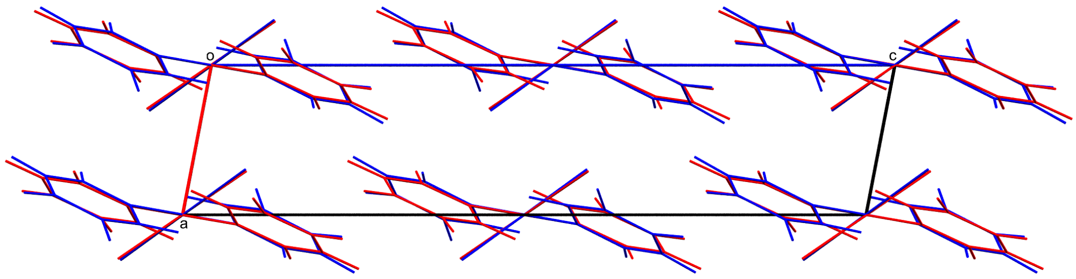

Figure S5
CuCP: overlay of the final structure from a user-controlled Rietveld refinement (red) and the published single crystal structure (after transformation from $P2_1/n$ to $P2_1/c$, blue). View along [010].

S7. Pawley fits of DFQ, DCQ and DHDCQ

The unit cells resulting from the Rietveld refinements of DFQ (Fig. 5, Table S5, Table S17), DCQ (Fig. 6, Table 5, Table S17) and DCDHQ (model B1Z1, Fig. 9, Table 8) were compared to the Pawley fits for the same powder data. The cells dimensions of DFQ and DCQ from the Pawley fit (Table S16) are in good agreement with the Rietveld refinements of those structures and the corresponding difference curves (Fig. S6 and Fig. S7) are quite flat. The difference curve of the Pawley fit of DCDHQ (Fig. S8) is less smooth than those of DFQ or DCQ, and indicates that the sample may contain traces of other phases.

Table S16
Pawley fits of DFQ, DCQ and DCDHQ.

Model	DFQ (B)	DCQ	DHDCQ (B1Z1)
Space group	$P2_1/c$	$P\overline{1}$	$P\overline{1}$
Z	2	1	1
R_{exp} / %	1.276	1.280	1.280
R_{wp} / %	5.160	6.759	9.946
R_{wp}' / %	9.018	12.249	17.679
GoF	4.045	5.279	7.770
V/Z / Å^3/mol	358.54	358.33	361.44
a / Å	14.2113(12)	3.7715(13)	3.8492(71)
b / Å	3.76353(13)	6.4745(20)	6.7222(21)
c / Å	13.6958(18)	15.7833(73)	15.6764(41)
α / °	90	93.695(33)	87.476(15)
β / °	102.595(15)	92.154(35)	91.238(51)
γ / °	90	100.814(51)	74.742(53)

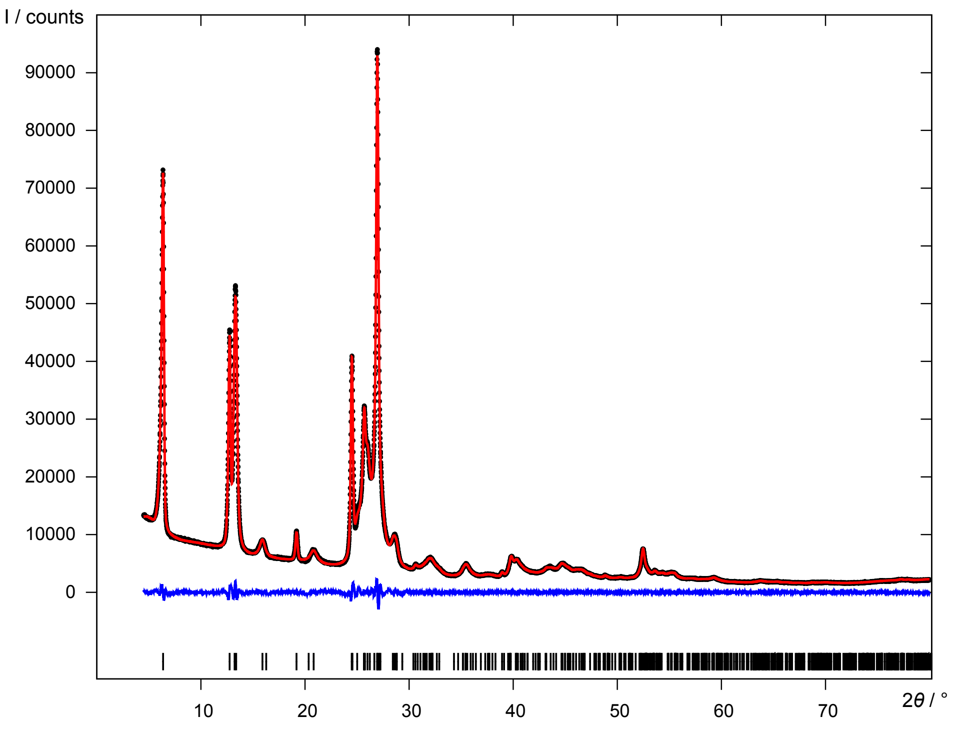

Figure S6
Pawley fit of DFQ ($P2_1$/c, Z=2, model B). Experimental X-ray powder diagram (black dots), simulated diagram of refined cell (red line), difference curve (blue line) and reflection positions (black bars).

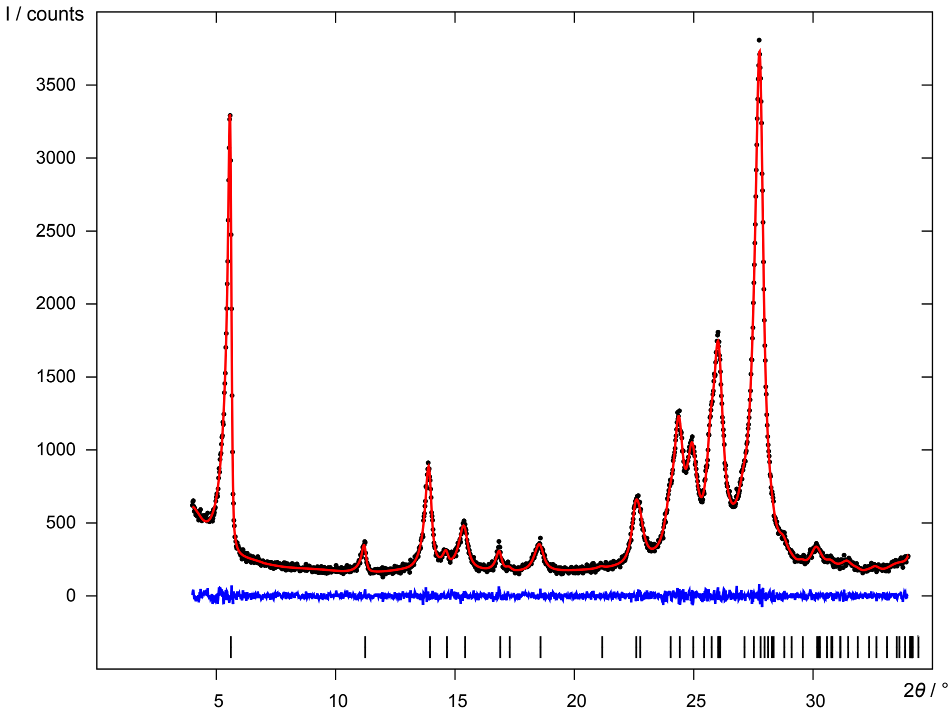

Figure S7
Pawley fit of DCQ ($P\bar{1}$, Z=1). Experimental X-ray powder diagram (black dots), simulated diagram of refined cell (red line), difference curve (blue line) and reflection positions (black bars).

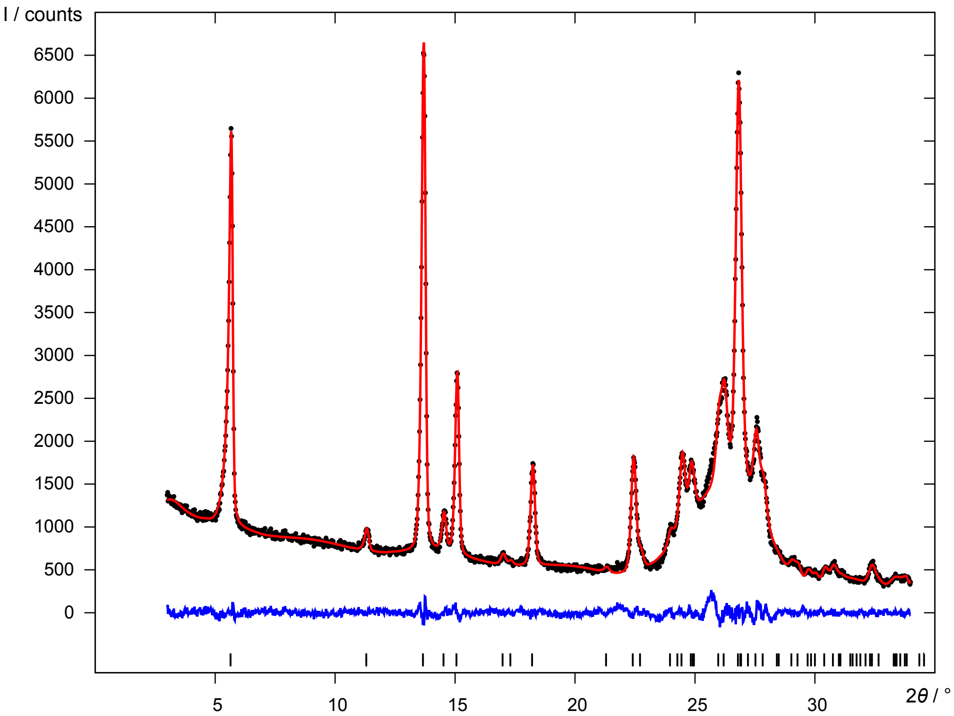

Figure S8
Pawley fit of DCDHQ ($P\bar{1}$, Z=1, model B1Z1). Experimental X-ray powder diagram (black dots), simulated diagram of refined cell (red line), difference curve (blue line) and reflection positions (black bars).

S8. Experimental details of the crystal structure determinations of DFQ and DCQ

Table S17
Experimental details of the crystal structure determinations of DFQ and DCQ (CIF file in the supporting information)

	DFQ	DCQ
Crystal data		
Chemical formula	$C_{20}H_{10}F_2N_2O_2$	$C_{20}H_{10}Cl_2N_2O_2$
M_r	348.30	381.20
Crystal system, space group	Monoclinic, $P2_1/c$	Triclinic, $P\bar{1}$
Temperature (K)	300	300
a, b, c (Å)	14.2169 (17), 3.7679 (2), 13.7214 (15)	3.7718 (11), 6.4792 (18), 15.774 (5)
α, β, γ (°)	90, 102.298 (14), 90	93.74 (3), 92.19 (2), 100.918 (19)
V (Å^3)	718.16 (13)	377.20 (19)
Z	2	1
Radiation type	Cu $K\alpha_1$, λ = 1.54056 Å	Cu $K\alpha_1$, λ = 1.54056 Å
Data collection		
Diffractometer	STOE STADI-P	STOE STADI-P
Specimen mounting	Prepared between polymer films	Prepared between polymer films
Data collection mode	Transmission	Transmission
Scan method	Continuous	Continuous
2θ values (°)	$2\theta_{min}$=4.50 $2\theta_{max}$=79.99 $2\theta_{step}$=0.01	$2\theta_{min}$=4.00 $2\theta_{max}$=33.98 $2\theta_{step}$=0.02
Refinement		
R factors and goodness of fit	R_p=0.054, R_{wp}=0.065, R_{exp}=0.015, $R(F)$=0.026, χ^2=19.378	R_p=0.040, R_{wp}=0.052, R_{exp}=0.046, $R(F)$=0.191, χ^2=1.288
No. of data points	7550	1500
No. of parameters	104	111
No. of restraints	61	61
H-atom treatment	Only H-atom coordinates refined	Only H-atom coordinates refined

Computer programs: *FIDEL-GO*, *WinX^{POW}* (STOE & Cie, 2006), *TOPAS Academic* (Coelho, 2009), *Mercury* (Macrae *et al.*, 2008).

References

Cheary, R. W. & Coelho, A. A. (1998). *J. Appl. Cryst.* **31**, 851–861.

Coelho, A. A., (2009). *TOPAS-Academic*. Version 4.2. Coelho Software, Brisbane, Australia.

Huang, R., Wang, C., Wang, Y. & Zhang, H. (2018). *Advanced Materials*, **30**, 1800814.

Macrae, C. F., Bruno, I. J., Chisholm, J. A., Edgington, P. R., McCabe, P., Pidcock, E., Rodriguez-Monge, L., Taylor, R., Streek, J. v. d. & Wood, P. A. (2008). *J. Appl. Cryst*, **41**, 466–470.

Pawley, G. S. (1981). *J. Appl. Cryst.* **14**, 357–361.

Spek, A. L. (2009). *Acta Cryst.* D**65**, 148–155.

STOE & Cie, (2006). *WinXPOW Version 2.15*. STOE & Cie GmbH, Darmstadt, Germany.
